# Fish consumption, omega-3 fatty acids, and risk of developing rheumatoid arthritis: a systematic review and meta-analysis

**DOI:** 10.1016/j.ero.2026.100272

**Published:** 2026-11-07

**Authors:** Anna-Maria Lampousi, Tam Lac, Marina Dehara, Lars Alfredsson, Daniela Di Giuseppe

**Affiliations:** 1 Division of Clinical Epidemiology, Department of Medicine Solna, Karolinska Institutet, Stockholm, Sweden; 2 Department of Molecular Medicine and Surgery, Karolinska Institutet, Stockholm, Sweden; 3 Institute of Environmental Medicine, Karolinska Institutet, Stockholm, Sweden; 4 Center for Occupational and Environmental Medicine, Region Stockholm, Stockholm, Sweden

## Abstract

**Objectives:**

Omega-3 fatty acids, primarily found in fish, have been linked to a reduced risk of rheumatoid arthritis (RA), but findings are inconsistent. We conducted a systematic review and meta-analysis to examine the associations between fish consumption, omega‑3 fatty acids, and RA risk.

**Methods:**

We searched Medline, Embase, and the Cochrane Library from inception to September 2025 for studies examining fish consumption, omega-3 fatty acid intake (dietary or supplemental), or circulating omega-3 levels in relation to RA risk. Random-effects models were used to estimate pooled risk ratios (RRs) and 95% CIs. Evidence certainty was assessed using the Grading of Recommendations, Assessment, Development and Evaluations framework. The protocol was registered in International Prospective Register of Systematic Reviews (CRD420251136451).

**Results:**

Of 4388 screened records, 13 cohort and 7 case-control studies were eligible for meta-analysis. Fish consumption was associated with a lower risk of RA, with a nonlinear dose-response relationship. Intake of 2 to 3 servings/wk was associated with the lowest risk (RR 0.76; 95% CI: 0.64-0.90 at 2.5 servings/wk), with attenuation at higher intakes (RR 0.97; 95% CI: 0.66-1.43 at 5.5 servings/wk). Similarly, higher dietary omega‑3 intake was associated with lower RA risk (per 1 g/d: RR 0.91; 95% CI: 0.85-0.97). No associations were observed for omega‑3 supplementation (RR 1.09; 95% CI: 0.85-1.40) or circulating omega‑3 levels (RR 0.94; 95% CI: 0.74-1.18); however, these findings were inconsistent and of very low certainty.

**Conclusions:**

Evidence suggests that higher fish consumption and intake of dietary omega-3 fatty acids are associated with a lower risk of RA. However, evidence from biomarker studies and supplementation remains limited.

**WHAT IS ALREADY KNOWN ON THIS TOPIC**

• Dietary factors may influence the risk of rheumatoid arthritis (RA), with particular interest in fish and omega‑3 fatty acids because of their anti-inflammatory properties.
• Previous studies and meta-analyses have reported inconsistent evidence regarding their role in RA prevention.

**WHAT THIS STUDY ADDS**

• Higher fish consumption is associated with a lower risk of RA, with the strongest associations observed at 2 to 3 servings/wk.
• Dietary omega‑3 fatty acid intake is inversely associated with RA risk per 1 g/d increase.
• Evidence from circulating biomarkers and supplementation also suggests an inverse association, although findings remain limited and less certain.

**HOW THIS STUDY MIGHT AFFECT RESEARCH, PRACTICE OR POLICY**

• These findings support current recommendations encouraging regular fish consumption as part of a healthy diet and suggest a potential role in RA prevention.
• Further research is needed to determine whether omega‑3 supplementation confers additional benefits, particularly among high-risk individuals.

Alt-text: Unlabelled box dummy alt text

## INTRODUCTION

Rheumatoid arthritis (RA) is an autoimmune condition marked by persistent inflammation of the synovial joints [1]. It is commonly classified into seropositive and seronegative subtypes, depending on the presence of rheumatoid factor (RF) and/or anticitrullinated protein antibodies (ACPAs) [2]. Seropositive RA, which accounts for the majority of cases, is typically preceded by a preclinical phase marked by detectable autoantibodies before the onset of clinical symptoms [3]. The development of RA is strongly influenced by genetic susceptibility, particularly the presence of shared epitope (SE) alleles within the *HLA-DRB1* gene [4]. Environmental factors interact with genetic predisposition to increase disease risk, with smoking representing one of the most well-established risk factors [5]. Diet has also gained attention because of its role in modulating systemic inflammation and immune responses [[6], [7], [8], [9]].

Fish consumption, particularly fatty fish rich in long-chain omega-3 fatty acids, has been hypothesised to reduce the risk of RA because of its anti-inflammatory properties. Experimental and clinical evidence indicates that omega-3 fatty acids influence inflammatory pathways relevant to RA, including cytokine production and eicosanoid synthesis [10]. However, epidemiological findings remain inconsistent. Earlier meta-analyses of observational studies suggested a potential inverse, nonlinear association between fish consumption and RA risk [11,12]. In contrast, a more recent meta-analysis restricted to prospective studies found no association with total fish intake and only a possible inverse association for fatty fish, with low certainty of evidence [13]. Similarly, meta-analysis of dietary omega-3 fatty acid intake has not demonstrated a clear association with RA risk, with very low certainty reflecting limited sample sizes and heterogeneity [13].

Since these analyses, additional studies have been published, potentially strengthening the evidence base. Integration of objective biomarker-based measures of omega-3 fatty acids and differentiation between specific omega-3 fatty acids may further strengthen the evidence. Potential effect modification by smoking also remains understudied. It is also unclear whether fish consumption or omega-3 fatty acids influence the development of RA-related autoantibodies or the progression from the preclinical to the clinical phase of disease.

This study aims to clarify the role of fish consumption and omega-3 fatty acids in the risk of developing RA by integrating evidence from dietary intake, supplementation, and biomarker-based measures of omega-3 fatty acids.

## METHODS

This study was reported in accordance with the Preferred Reporting Items for Systematic Reviews and Meta-Analyses guidelines [14]. The study protocol was registered in the International Prospective Register of Systematic Reviews (registration number CRD420251136451).

### Search strategy and selection criteria

A literature search was performed in Medline, Embase, and Cochrane Library from inception to September 26, 2025, without language restrictions, in collaboration with librarians at the Karolinska Institutet University Library. The complete search strategies for each database are provided in Supplementary Tables S1 to S3.

Eligible studies included observational designs (cohort, case-cohort, case-control, nested case-control, and cross-sectional studies) and randomised controlled trials (RCTs) that examined the association between fish consumption, omega-3 fatty acid intake (dietary or supplemental), or circulating omega-3 fatty acid levels and clinical or preclinical RA. Preclinical RA was defined as the presence of RF and/or ACPAs in the absence of a clinical RA diagnosis. Conference abstracts, editorials, interviews, letters, and animal studies were excluded. In addition, the reference lists of included articles and relevant systematic reviews and meta-analyses were manually screened to identify further eligible studies. All records were deduplicated using Covidence [15], supplemented by manual identification of remaining duplicates. Using the same tool, 2 authors (A-ML and TL) independently screened titles and abstracts of all unique records and subsequently assessed the full texts of potentially eligible studies. Discrepancies were resolved through discussion, with involvement of a third author (DDG) when necessary.

### Data extraction

Data were independently extracted by 2 authors (A-ML and TL). The following information was collected from each included study: first author, year of publication, journal, country, and study name (if applicable); study design; sample size; number of exposed and unexposed cases, and exposed and unexposed controls or noncases; participant characteristics (including sex, age, and smoking status at baseline); and details of the exposure, including fish consumption (frequency and quantity), type of fish (total or fatty), and omega-3 fatty acid measures (dietary intake, supplementation, or circulating levels, including quantity, frequency, or yes/no). Additional extracted data included comparator definition; outcome (RA or preclinical RA); SE status; RF and/or ACPA positivity at diagnosis; methods used for exposure and outcome assessment; response and attrition rates; duration of follow-up and person-time (where applicable); the most fully adjusted relative effect estimates with corresponding 95% CIs; covariates included in adjusted models; and funding sources. When multiple publications were based on overlapping study populations, only the most recent article was included in meta-analysis.

### Risk-of-bias assessment

Two authors (A-ML and TL) independently assessed the risk of bias in the included studies using the Risk Of Bias In Nonrandomized Studies of Exposure (ROBINS-E) tool for cohort and nested case-control studies [16], and the Risk of Bias in Randomized Trials (RoB 2) tool for RCTs [17]. Disagreements were resolved by adopting the more conservative (higher risk) rating. The ROBINS-E tool evaluates 7 domains of bias: confounding; measurement of exposure; selection of participants into the study or analysis; postexposure interventions; missing data; outcome measurement; and selection of the reported result. Each domain is rated as low risk, some concerns, or high risk of bias. Studies that did not adjust for at least one indicator of socioeconomic status or lifestyle factors were classified as having some concerns due to confounding, provided age and sex were adjusted for when applicable; failure to adjust for ≥2 of these factors was considered high risk. The RoB 2 tool evaluates 5 domains of bias in RCTs: the randomisation process; deviations from intended interventions; missing outcome data; outcome measurement; and selection of the reported result. For both ROBINS-E and RoB 2, the overall risk of bias for each study was determined by the domain with the highest level of bias. The current version of ROBINS-E does not apply to case-control studies and was therefore not used for such designs. Case-control studies were considered to be at high risk of bias, primarily due to the potential for differential misclassification of dietary exposures. Cross-sectional studies were considered to be at critical risk of bias because temporality between exposure and outcome cannot be established, and were therefore included for narrative synthesis only and not meta-analysed.

### Statistical analysis

Pooled risk ratios (RRs) and 95% CIs for RA were estimated for the highest vs lowest categories of fish consumption and dietary omega-3 intake, for any vs no omega-3 supplementation, and per 1% increment in circulating omega-3 fatty acid levels using random-effects models on the natural logarithm scale. Between-study variance was estimated using the restricted maximum likelihood method. Linear dose-response meta-analyses were conducted to evaluate fish consumption and dietary omega-3 intake as continuous exposures. For studies that did not report dose-response estimates, study-specific slopes and corresponding 95% CIs were derived using the method described by Greenland and Longnecker [18], based on the natural logarithm of the reported RRs across exposure categories. This approach requires information on the number of cases, person-time or number of total participants, and the mean or median exposure levels with corresponding RRs and 95% CIs across at least 3 exposure categories. When this information was not reported across exposure categories, it was approximated by dividing the total number evenly across categories. For exposure categories reported as ranges, midpoints were assigned. For open-ended categories, the lower bound was assumed to be zero for the lowest category, and for the highest category, an interval width equivalent to the adjacent category was assumed. Linear trends were expressed as RRs per 1 serving/wk for fish consumption, per 1 g/d for dietary omega-3 fatty acids, and per 1% increment for circulating omega-3 fatty acids. Whenever results were reported as increment per 1 SD, information on the SD was retrieved from the papers [[19], [20], [21]] and harmonised to 1% increment. For fish consumption, 1 serving was defined as 100 g. Nonlinear dose-response relationships were additionally examined using random-effects restricted cubic spline models with 3 knots placed at the 10th, 50th, and 90th percentiles of the exposure distribution [22]. Studies reporting only linear estimates were not included in the nonlinear analyses. When estimates for total fish consumption were not available, those for fatty fish were used (n = 2).

Heterogeneity across studies was assessed using Cochran’s Q test and quantified with the I² statistic, with values >50% indicating substantial heterogeneity. Subgroup analyses were not performed, as heterogeneity was observed in only one meta-analysis, which included a limited number of studies (n = 3). Metaregression was conducted within this meta-analysis to explore the proportion of smokers in each study as a potential source of heterogeneity. Sensitivity analyses were conducted by excluding studies at high risk of bias. When serostatus data were available, separate analyses were conducted for ACPA- and RF-positive, or ACPA-positive–only RA (when combined data were unavailable), and seronegative RA. Small-study effects were evaluated using Egger’s test in meta-analyses including ≥10 studies, and potential publication bias was assessed by visual inspection of funnel plots.

All statistical analyses were performed using Stata Statistical Software, Release 16 (StataCorp).

### Certainty of evidence assessment

The certainty of evidence for each meta-analysis was assessed using the Grading of Recommendations Assessment, Development and Evaluation framework [23]. In line with this approach, evidence from meta-analyses was initially considered to have high certainty, equivalent to 4 points [16]. The certainty of evidence was subsequently downgraded by 1 or 2 points based on the presence of concerns related to risk of bias, inconsistency, imprecision, indirectness, or publication bias. Certainty could be upgraded in the presence of a large effect size (RR < 0.5 or RR > 2) (+ 1 or 2 points), evidence of a dose-response gradient (+1 point), or when all plausible sources of bias were considered likely to attenuate the observed association (+1 point). The overall certainty of evidence was ultimately classified as very low, low, moderate, or high.

## RESULTS

After screening 4388 records, 34 studies met the eligibility criteria (Fig 1). Records excluded following full-text screening, along with reasons for exclusion, are detailed in Supplementary Table S4. Characteristics of the included studies are summarised in Supplementary Tables S5 and S6. The included studies were evenly distributed between Europe and the United States (n = 14 each), with the remainder conducted in Asia (n = 6). Risk of bias among cohort and nested case-control studies varied by exposure type; studies on fish consumption and dietary omega-3 fatty acids were primarily rated as having some concerns, whereas those assessing circulating omega-3 fatty acids were more often at low risk of bias (Supplementary Figs S1-S3). This discrepancy is mainly due to greater susceptibility to measurement error in dietary studies, which rely on self-reported assessments. The only RCT, reported across 2 publications from the VITamin D and OmegA-3 TriaL (VITAL) (Supplementary references 70-71), was rated as having low risk of bias. Fourteen eligible studies were excluded from the meta-analysis due to cross-sectional design (Supplementary references 60-65), overlapping data with other cohorts (Supplementary references 66-69), analysis of the VITAL trial data (Supplementary references 70-71), or study of preclinical RA (Supplementary references 72-73) (Supplementary Table S6). Consequently, 13 cohort studies (including 3 nested case-control studies) and 7 case-control studies were included in the meta-analyses (Fig 1).

**Figure 1:**
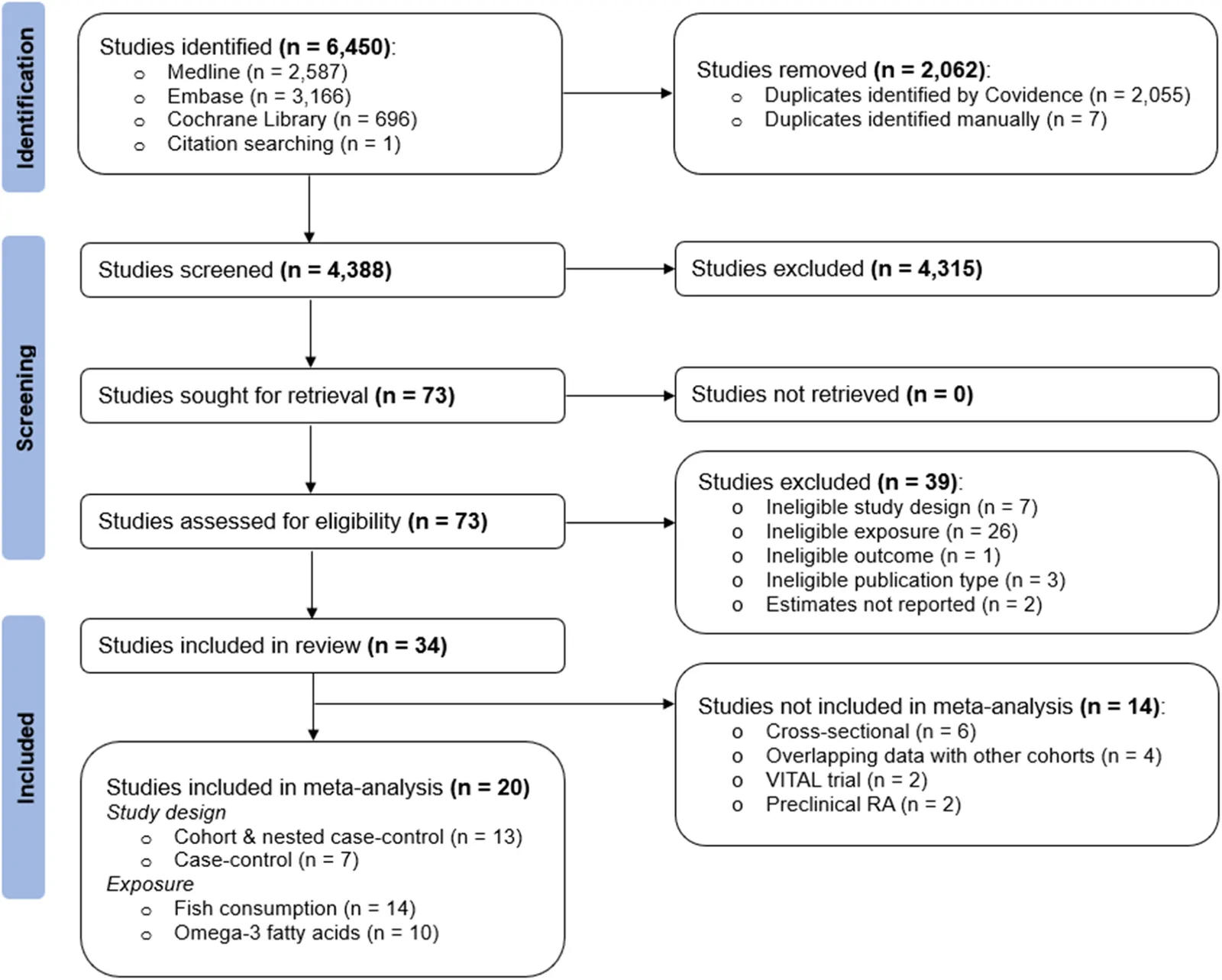
Flow diagram of study selection. RA, rheumatoid arthritis; VITAL, VITamin D and OmegA-3 TriaL.

### Fish consumption

A total of 6 cohort [[24], [25], [26], [27], [28], [29]] and 6 case-control [[30], [31], [32], [33], [34], [35]] studies, comprising 4132 RA cases, were included in the high vs low analysis of fish consumption. Reported intakes ranged from 0 to 17.5 servings/wk. Higher fish consumption was associated with a lower risk of RA (RR: 0.88; 95% CI: 0.81-0.96; I² = 0%) (Fig 2), with no evidence of small study effects (Egger’s test *P* = .5106). However, visual inspection of the funnel plot suggested a possible absence of small studies reporting positive associations (Supplementary Fig S4). Also, exclusion of studies with high risk of bias led to an attenuation of the estimate (RR: 0.90; 95% CI: 0.81-1.00, Supplementary Fig S5). No linear dose-response relationship was observed in the analysis of 7 cohort [[24], [25], [26], [27], [28], [29],^36^] and 5 case-control [[32], [33], [34], [35],^37^] studies (n = 8838 RA cases), with an RR of 1.00 (95% CI: 0.98-1.02; I² = 0%) per 1 serving/wk increase (Fig 2). However, a nonlinear dose-response relationship between fish consumption and RA risk was observed (*P* = .0006). Intake of 2 to 3 servings/wk was associated with the lowest risk. As an illustration, 2.5 servings/wk was associated with a 24% lower risk of RA (RR: 0.76; 95% CI: 0.64-0.90) compared with no consumption, whereas higher intakes showed attenuation of the association, reaching an RR of 0.97 (95% CI: 0.66-1.43) at 5.5 servings/wk (Fig 3). Sensitivity analyses excluding case-control studies and cohort studies at high risk of bias yielded results consistent with the main analyses (Supplementary Fig S5). The certainty of evidence for both high vs low and continuous analyses was rated as moderate (Supplementary Table S7). Analyses stratified by RA serostatus, based on 3 cohort [24,28,29] and 1 case-control [34] studies comparing high vs low fish intake, yielded an RR of 0.88 (95% CI: 0.74-1.05; I² = 0%) for seropositive RA and 0.97 (95% CI: 0.66-1.43; I² = 36.5%) for seronegative RA (Supplementary Fig S6). Only one cross-sectional study examined fish consumption in relation to RA, reporting an increased risk when comparing highest vs lowest intake [38]. No studies assessed the association between fish consumption and the development of RA-related autoantibodies or progression from seropositivity to RA.

**Figure 2:**
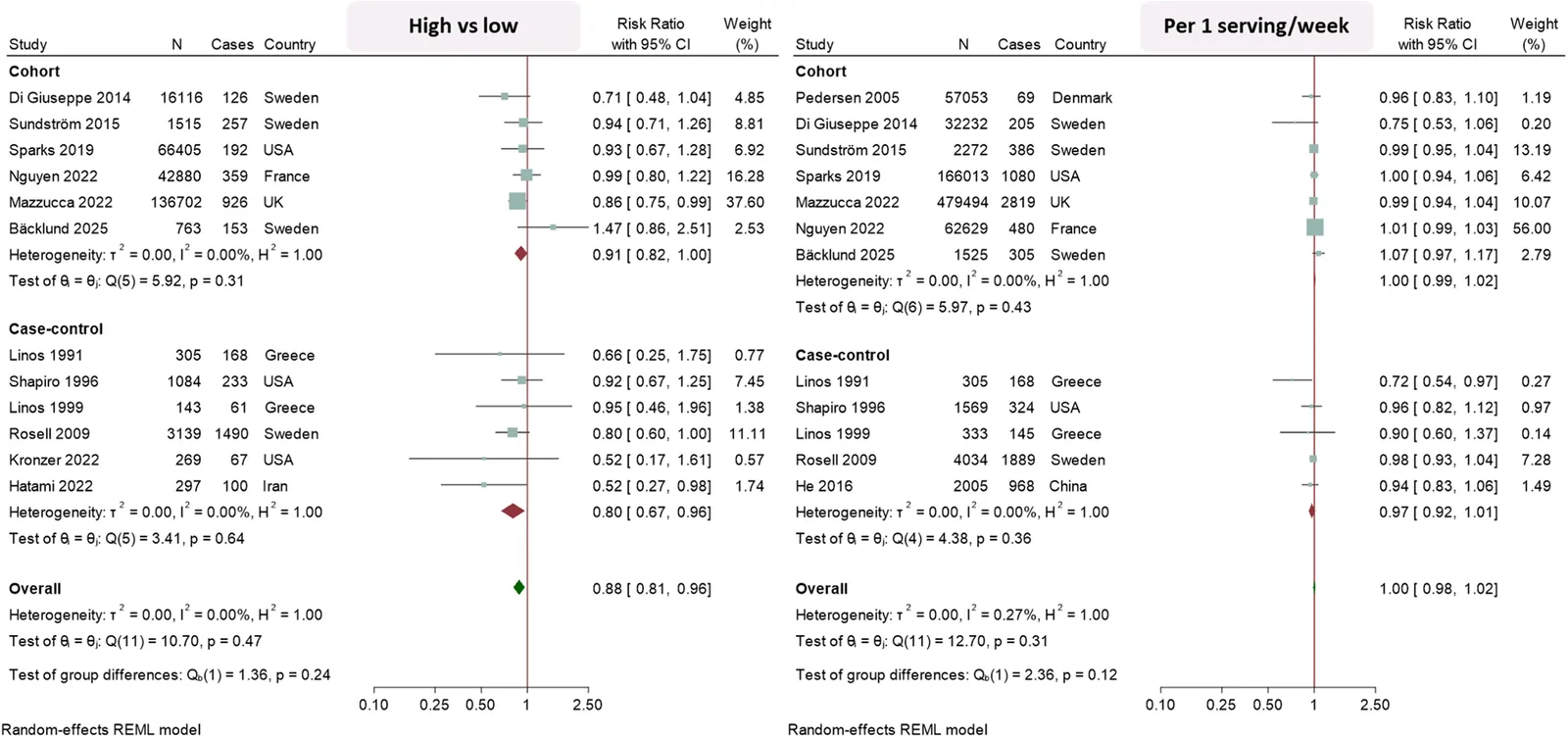
Forest plots of risk ratios for rheumatoid arthritis comparing high vs low fish consumption and per 1 serving/wk increase. REML=Restricted Maximum Likelihood.

**Figure 3:**
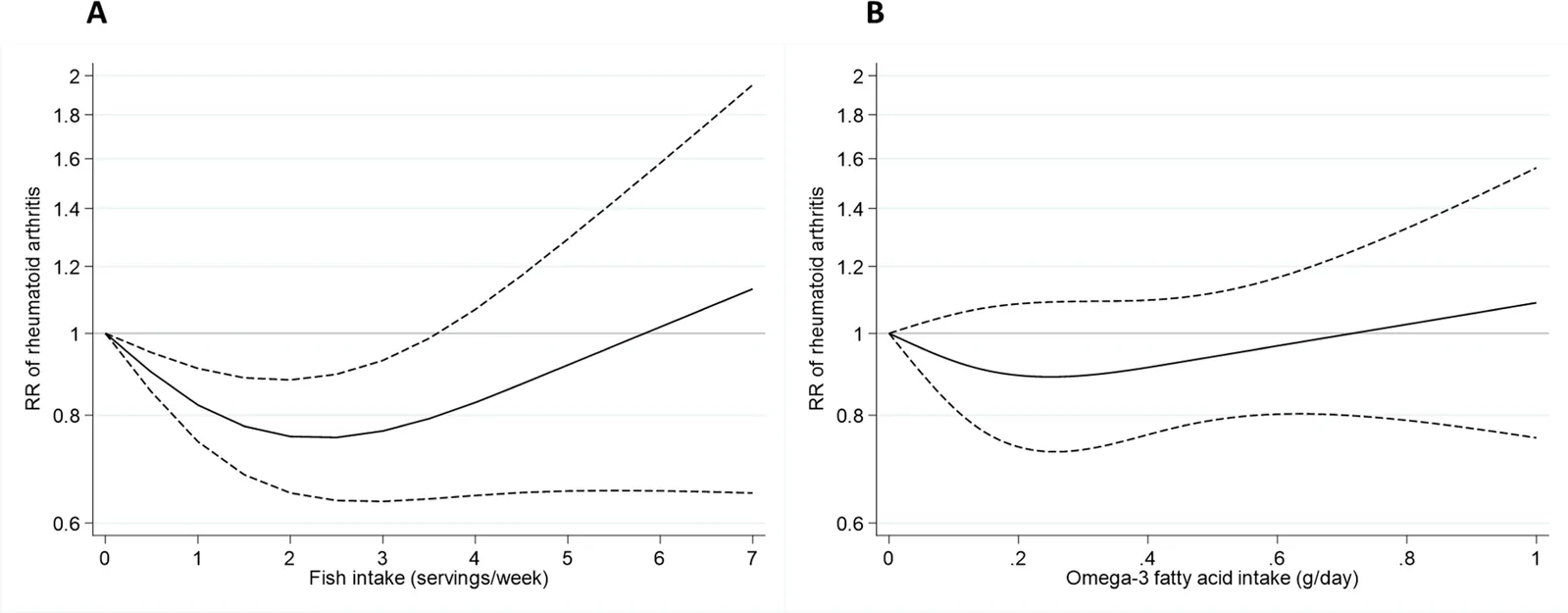
Nonlinear dose-response meta-analyses for the association between (A) fish consumption (*P*_nonlinearity_ = .0006) and (B) dietary omega-3 fatty acid intake (*P*_nonlinearity_ = .2366), and risk of rheumatoid arthritis. RR, risk ratio.

### Dietary omega-3 fatty acids

A total of 4 cohort [25,29,39,40] and 1 case-control [35] studies, comprising 2138 RA cases, were included in the high vs low analysis of dietary omega‑3 fatty acid intake. Reported intakes ranged from 0 to 1.85 g/d. Higher intake was not associated with the risk of RA (RR: 0.94; 95% CI: 0.83-1.07; I² = 21.9%) (Fig 4). However, a linear dose-response relationship was observed in the analysis of 5 cohort [19,25,29,39,40] and 1 case-control [35] studies (n = 6347 RA cases), with an RR of 0.91 (95% CI: 0.85-0.97; I² = 0%) per 1 g/d increase in intake (Fig 4). There was no evidence of a nonlinear dose-response relationship (*P* = .2366) (Fig 3). Sensitivity analyses excluding the case-control study and cohort studies at high risk of bias yielded stronger effect estimates; however, CIs were wide: the RR was 0.80 (95% CI: 0.64-1.01; I² = 0%) for the high vs low comparison and 0.73 (95% CI: 0.44-1.21; I² = 41.9%) per 1 g/d increase (Supplementary Fig S7). The certainty of evidence was rated as low for the high vs low analysis and moderate for the continuous analysis (Supplementary Table S7).

**Figure 4:**
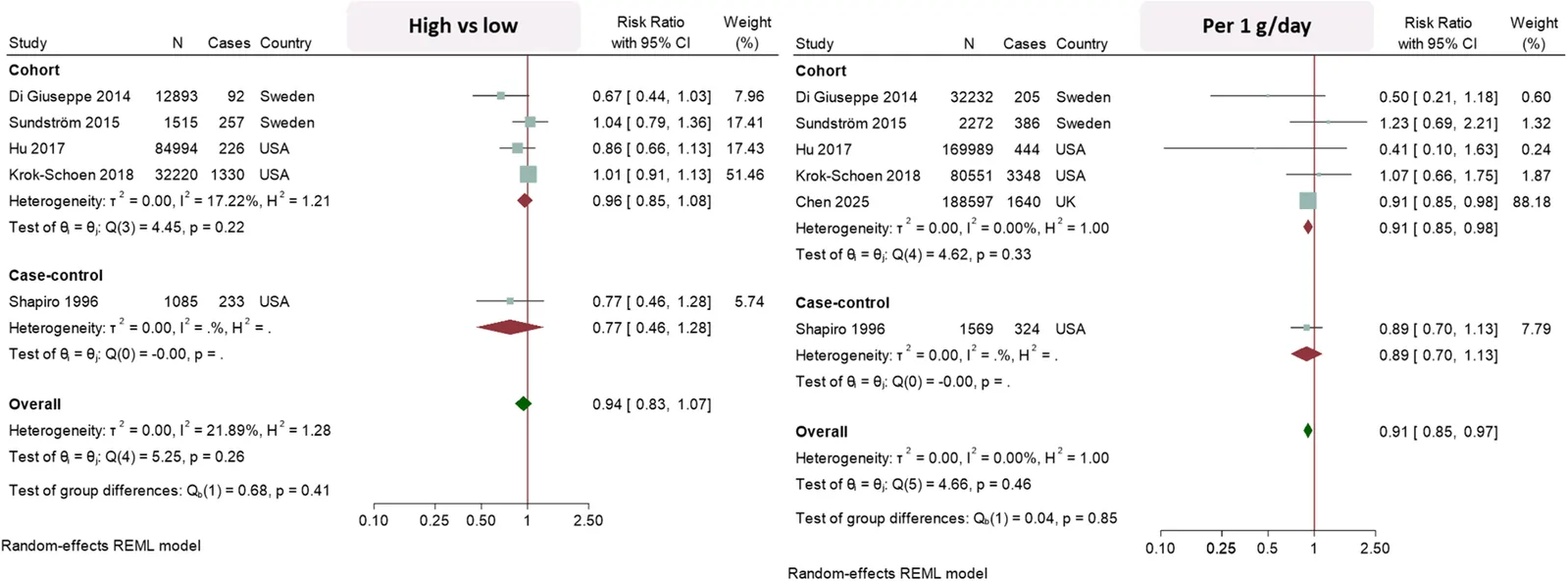
Forest plots of risk ratios for rheumatoid arthritis comparing high vs low dietary omega-3 fatty acid intake and per 1 g/d increase. REML= Restricted Maximum Likelihood.

Among studies evaluating individual omega‑3 fatty acids, 1 study reported inverse associations for all fatty acids except alpha-linolenic acid (ALA) per 1 SD increase [19], whereas another study found no associations when comparing high vs low intakes [40]. One large cross-sectional study based on the National Health and Nutrition Examination Survey (NHANES) data reported an inverse association between high vs low dietary omega‑3 intake and RA [41]. Another analysis on a subset of the same dataset found inverse associations specifically for docosahexaenoic acid (DHA), but not for other omega‑3 fatty acids [42]. In contrast, 2 studies based on the Korean NHANES data did not observe any association between dietary omega‑3 intake and RA [43,44].

### Omega-3 supplementation

Two case-control studies involving 342 cases were included in the meta-analysis comparing any vs no omega‑3 supplementation in relation to RA risk [31,34]. No association was observed (RR: 1.09; 95% CI: 0.85-1.40; I² = 0%) (Supplementary Fig S8), and the certainty of this evidence was rated as very low. In contrast, results from the VITAL trial, which evaluated omega‑3 supplementation vs placebo over 5 years, suggested a potential protective effect on RA development [45]. Reduced risk estimates were observed both at the end of the intervention (RR: 0.66; 95% CI: 0.37-1.21) and 2 years postintervention (RR: 0.64; 95% CI: 0.39-1.06), although CIs were wide. Similarly, in the Studies of the Etiology of Rheumatoid Arthritis (SERA) cohort, omega‑3 supplementation (any vs none) was associated with a lower risk of developing ACPA positivity (RR: 0.11; 95% CI: 0.02-0.61) [46].

### Circulating omega-3 fatty acids

The analysis of circulating omega‑3 fatty acids included 3 cohort studies comprising 730 RA cases [20,21,47]. No association was observed between a 1% increase in circulating omega‑3 levels and RA risk (RR: 0.94; 95% CI: 0.74-1.18; I² = 58.6%) (Supplementary Fig S9). Heterogeneity was substantial, and the proportion of smokers across cohorts was identified as a potential effect modifier in the metaregression analysis (*P* = .0456). Specifically, 2 studies with smoking prevalence ≤10% suggested inverse associations with higher circulating omega‑3 levels, whereas 1 study with smoking prevalence >25% showed no association (Supplementary Fig S10). Exclusion of 1 study judged to be at high risk of bias resulted in an RR of 1.01 (95% CI: 0.76-1.33; I² = 69.7%) (Supplementary Fig S11). Overall, the certainty of evidence for the association between circulating omega‑3 fatty acids and RA risk was rated as very low.

Among the included studies, findings on individual omega‑3 fatty acids were inconsistent: 1 study reported an inverse association for DHA [20], whereas another found no association for any individual fatty acids [47]. The third study, conducted within the SERA cohort, evaluated progression from ACPA positivity to RA. Higher levels of all omega‑3 fatty acids except ALA were compatible with a reduced risk of progression, although only docosapentaenoic acid reached statistical significance [21]. Two additional studies from the same cohort examined circulating omega‑3 fatty acids in relation to preclinical RA. One reported a reduced risk of developing ACPA [46], whereas the other, stratified by SE status, found inverse associations with ACPA and high-titre RF among individuals carrying the SE allele, but not among noncarriers [48]. A single cross-sectional study among Korean women reported inverse associations between higher circulating ALA and eicosapentaenoic acid (EPA) levels and RA, whereas no associations were observed for other omega‑3 fatty acids [49].

## DISCUSSION

In this meta-analysis of 13 prospective cohort and 7 case-control studies, higher fish consumption and dietary omega-3 fatty acid intake were associated with reduced RA risk, with evidence graded as moderate certainty. The association with fish intake appeared nonlinear, with stronger associations observed for 2 to 3 servings/wk, followed by attenuation at higher intakes, whereas omega‑3 fatty acid intake demonstrated a more linear relationship. This pattern may reflect a balance between the potential benefits of omega‑3 fatty acids and exposure to environmental contaminants present in fish, which could attenuate the inverse associations at higher levels of consumption. Additional support for an inverse association of omega-3 fatty acids was provided by studies of supplementation and circulating levels; however, the evidence was limited and inconsistent, with lower overall certainty.

Our findings of an inverse association between fish consumption and RA risk are consistent with prior meta-analyses [[11], [12], [13]], and extend the evidence base by including more recent studies, thereby increasing overall certainty. It has been suggested that this association may be driven primarily by fatty fish [13], which is the main dietary source of long-chain omega‑3 fatty acids. In our meta-analysis, most studies assessed total fish intake, with only 2 reporting estimates specifically for fatty fish [26,34]. These estimates were consistent with those for total fish, although more precise. Notably, among the few cohorts reporting both measures, 1 observed no association for either total or fatty fish [27], whereas another reported an association restricted to fatty fish [36]. Although limited, these findings are compatible with the hypothesis that fatty fish may be particularly relevant due to its omega‑3 content. It is also not to be excluded that the observed inverse association between fish consumption and risk of RA may be related to other nutrients, like vitamin D [9]. Consistently, our meta-analysis demonstrated an inverse association between dietary omega‑3 fatty acid intake and RA risk. This expands upon an earlier meta-analysis, which did not detect a significant association [13]. In contrast, analyses of circulating omega-3 fatty acids did not show a clear overall association. However, differences in smoking prevalence emerged as a potential effect modifier, with higher smoking rates potentially attenuating associations. Smoking is a well-established risk factor for RA and may obscure the potential inverse association of omega‑3 fatty acids. Notably, evidence from the SERA study suggests that omega‑3 fatty acids may be more relevant in earlier stages of disease development and play a role in autoimmunity, as higher circulating levels and supplementation were associated with lower risk of RA-related autoantibody positivity, especially in SE carriers [21,46,48]. Our analyses of fish intake likewise indicated that inverse associations may be confined to seropositive RA, although estimates were imprecise. Consistently, findings from the VITAL trial, the only RCT in this area and at low risk of bias, suggested a reduction in the incidence of autoimmune diseases, including RA, following 5 years of omega‑3 supplementation [45]. However, our meta-analysis of case-control studies did not provide consistent support, though the findings were of low certainty because it was limited to 2 studies (342 cases). Overall, evidence from supplementation and circulating omega‑3 biomarker studies remains limited and heterogeneous, and further research is needed to determine whether omega-3 supplementation confers additional benefit, particularly in individuals at higher risk of RA.

Besides possible effects on autoimmunity, omega‑3 fatty acids have well-established anti-inflammatory and inflammation-resolving properties. Long-chain omega‑3 fatty acids, particularly EPA and DHA, which are primarily found in fatty fish, compete with arachidonic acid for enzymatic conversion, leading to the production of less proinflammatory eicosanoids [50]. In addition, EPA and DHA serve as precursors to specialised proresolving mediators, such as resolvins and protectins, which actively promote the resolution of inflammation [51]. Consistent with these mechanisms, studies examining specific fatty acids more consistently reported associations for long-chain omega‑3 fatty acids. In contrast, ALA, a plant-derived shorter-chain omega‑3 fatty acid, must be converted in the body to EPA and DHA, but this conversion is limited in humans [52], which may explain weaker and less consistent findings. The EPA and DHA content of fish varies widely, ranging from ∼2.63 g per 100 g in Atlantic mackerel to as little as 0.01 g per 100 g in leaner species such as striped catfish [53].

### Strengths and limitations

Strengths of this systematic review include a comprehensive literature search supported by experienced librarians and a systematic assessment of certainty of evidence. We applied complementary analytic approaches, including high vs low comparisons and dose-response meta-analyses, to capture potentially complex associations. By integrating evidence across multiple study designs, we provide a comprehensive synthesis of the available literature.

This study also has some limitations. Although no language restrictions were applied, the search strategy was conducted using English-language terms, which may have led to the omission of relevant studies published in other languages. The number of included studies in meta-analyses was often <10, limiting our ability to assess publication bias. We did not perform subgroup analyses, as heterogeneity was generally low; the only analysis with detectable heterogeneity included too few studies to support meaningful exploration. Associations were generally stronger in case-control than cohort studies. Although this may reflect greater susceptibility to recall bias, it may also arise from differences in exposure assessment, as case-control studies capture diet closer to diagnosis, whereas exposures may change over longer follow-up in cohort studies. Nevertheless, sensitivity analyses excluding case-control studies and cohorts at higher risk of bias did not alter the findings. Although cohorts generally had a low risk of bias across most ROBINS‑E domains, the majority were judged as having ‘some concerns’ overall. This was mainly due to concerns in the exposure measurement domain of dietary studies, as the overall judgement is driven by the domain with the highest risk of bias. Moreover, we considered all case-control studies as high risk of bias without a detailed assessment, as a corresponding tool has not yet been released, which may have led to a stricter overall judgement. Finally, although studies spanned multiple regions, they were concentrated in a limited number of countries, particularly Sweden and the United States, which may limit generalisability. Differences in fish species, preparation methods, and broader dietary patterns across regions may influence both omega‑3 exposure and associated health effects.

## CONCLUSION

Evidence indicates that fish consumption is associated with a lower risk of RA, with strongest associations at 2 to 3 servings/wk. Consistently, higher dietary intake of omega‑3 fatty acids shows an inverse association with RA risk. Additional support from studies of circulating biomarkers and supplementation suggests an inverse association; however, this evidence remains limited, heterogeneous, and of lower certainty.

## CRediT authorship contribution statement

**Anna-Maria Lampousi:** Writing – original draft, Visualization, Methodology, Formal analysis, Data curation, Conceptualization. **Tam Lac:** Writing – review & editing, Data curation. **Marina Dehara:** Writing – review & editing. **Lars Alfredsson:** Writing – review & editing, Methodology, Funding acquisition, Conceptualization. **Daniela Di Giuseppe:** Writing – review & editing, Supervision, Methodology, Conceptualization.

## Competing interests

All authors declare they have no competing interests.

## Appendix Supplementary materials

Image, application 1

## Footnotes

Supplementary material associated with this article can be found in the online version at doi:10.1016/j.ero.2026.100272.
