# Supplementary material for: Fish consumption, omega-3 fatty acids, and risk of developing rheumatoid arthritis: a systematic review and meta-analysis
Source: EULAR Rheumatol Open. 2026 Nov 7;2(4):100272. doi: 10.1016/j.ero.2026.100272 (PMC13763020; doi:10.1016/j.ero.2026.100272)
Supplement: Supplementary file 1 [file mmc1.pdf]

SUPPLEMENTARY MATERIAL

Fish consumption, omega-3 fatty acids, and risk of developing rheumatoid arthritis: a systematic review and meta-analysis

Authors:
Anna-Maria Lampousi, Tam Lac, Marina Dehara, Lars Alfredsson, Daniela Di Giuseppe

Correspondence to:
Daniela Di Giuseppe
Division of Clinical Epidemiology, Department of Medicine Solna, Karolinska Institutet
K2 Medicin, Solna, K2 KEP Askling, 171 77 Stockholm, Sweden
daniela.digiuseppe@ki.se

Table of Contents

Supplementary Table 1. Search strategy for identification of relevant articles in Medline 3

Supplementary Table 2. Search strategy for identification of relevant articles in Embase 4

Supplementary Table 3. Search strategy for identification of relevant articles in Cochrane Library 6

Supplementary Table 4. Studies that were excluded after full text screening and reasons for exclusion 7

Supplementary Table 5. Characteristics of studies included in meta-analysis 8

Supplementary Table 6. Characteristics of eligible studies not included in meta-analysis 14

Supplementary Table 7. Certainty of evidence assessment using the GRADE system 20

Supplementary Figure 1. Risk of bias assessment of cohort and nested case-control studies on fish consumption using ROBINS-E, generated with the robvis tool 22

Supplementary Figure 2. Risk of bias assessment of cohort and nested case-control studies on dietary omega-3 fatty acids using ROBINS-E, generated with the robvis tool 23

Supplementary Figure 3. Risk of bias assessment of cohort and nested case-control studies on circulating omega-3 fatty acids using ROBINS-E, generated with the robvis tool 24

Supplementary Figure 4. Contour-enhanced funnel plot of risk ratios for rheumatoid arthritis comparing high versus low fish consumption 25

Supplementary Figure 5. Forest plots of risk ratios for rheumatoid arthritis comparing high versus low fish consumption and per 1 serving/week increase, after excluding studies at high risk of bias. 26

Supplementary Figure 6. Forest plots of risk ratios for seropositive and seronegative rheumatoid arthritis comparing high versus low fish consumption.. 27

Supplementary Figure 7. Forest plots of risk ratios for rheumatoid arthritis comparing high versus low dietary omega-3 fatty acid intake and per 1 g/day increase, after excluding studies at high risk of bias. 28

Supplementary Figure 8. Forest plot of risk ratios for rheumatoid arthritis comparing any versus no use of omega-3 fatty supplements.. 29

Supplementary Figure 9. Forest plot of risk ratios for rheumatoid arthritis per 1% increment in circulating omega-3 fatty acids. 30

Supplementary Figure 10. Meta-regression of risk ratios for rheumatoid arthritis per 1% increment in circulating omega-3 fatty acids with smoking prevalence within the cohort as effect modifier 31

Supplementary Figure 11. Forest plot of risk ratios for rheumatoid arthritis per 1% increment in circulating omega-3 fatty acids, after excluding studies at high risk of bias. 32

Supplementary references 33

Supplementary Table 1. Search strategy for identification of relevant articles in Medline

Interface: **Ovid MEDLINE(R) ALL** content coverage from 1946<br>Date of Search: September 26, 2025<br>Number of hits: 2587<br>Comment: In Ovid, two or more words are automatically searched as phrases; i.e. no quotation marks are needed	Field labels<br>• exp/ = exploded MeSH term<br>• / = non exploded MeSH term<br>• .ti,ab,kf. = title, abstract and author keywords<br>• adjx = within x words, regardless of order<br>• * = truncation of word for alternate endings<br>• ? = 0-1 letter/number<br>• # = 1 letter/number

#	Searches	Results
1	exp Seafood/	19335
2	exp Fatty Acids, Omega-3/	31564
3	exp Fish Oils/	35011
4	Fatty Acids, Unsaturated/	22754
5	exp Dietary Fats/	105083
6	Diet, High-Fat/	28209
7	Diet, Fat-Restricted/	4161
8	((algal or cod liver or fish or krill or marine or tuna) adj3 oil?).ti,ab,kf.	16848
9	(fatty acid* adj3 (polyunsaturat* or unsaturat*)).ti,ab,kf.	51973
10	(fatty acid* adj3 (n3 or n-3)).ti,ab,kf.	11937
11	(bilantin omega or conchol 36 or "eicosa e" or eicosapen or epaisdin or epanova or omega3 or omega-3 or omega forte or sakana).ti,ab,kf.	23936
12	((fatty acid* or fish) adj5 (consum* or diet* or feed* or intake or ingest*)).ti,ab,kf.	46482
13	(ala or dha or epa or pufa).ti,ab,kf.	94962
14	(alpha-linolenic acid* or docosahexaenoate or docosahexaenoic acid* or eicosapentaenoic acid* or icosapent or linolenate or timnodonic acid*).ti,ab,kf.	27516
15	(fish* product* or seafood? or sea-food? or shellfish*).ti,ab,kf.	21952
16	((diet* or food* or high* or low* or nutri*) adj5 (fat? or fatty)).ti,ab,kf.	174079
17	(fat? adj2 (alimentary or consum* or feed* or ingest* or intake)).ti,ab,kf.	18403
18	or/1-17	407884
19	Diet/	203036
20	Dietary Patterns/	2
21	(diet? or dietary or food?).ti,kf.	466186
22	((diet* or food*) adj3 (component* or consum* or expos* or factor* or group* or ingest* or intake* or pattern* or qualit*)).ab.	275502
23	or/19-22	693117
24	Risk/	129133
25	Risk Factors/	1050865
26	risk*.ti,ab,kf.	3465004
27	or/24-26	3813241
28	23 and 27	128392
29	18 or 28	509116
30	exp Arthritis, Rheumatoid/	133529
31	Arthritis/	38108
32	Rheumatic Diseases/	26098
33	Autoimmune Diseases/	61464

34	Rheumatoid Factor/	9477
35	Anti-Citrullinated Protein Antibodies/	821
36	(ACPA? or anti CCP or arthritis or arthritides or arthrosis deformans or autoimmune disease* or citrullinated peptide antibod* or citrullinated peptide autoantibod* or citrullinated protein autoantibod* or citrullinated protein antibod* or oligoarthritis or oligoarthritides or polyarthritis or polyarthritides or rheumatism or rheumarthritis or rheumatic disease* or rheumatoid factor*).ti,ab,kf.	343261
37	or/30-36	425512
38	29 and 37	3067
39	exp animals/ not humans.sh.	5378536
40	38 not 39	2587

Supplementary Table 2. Search strategy for identification of relevant articles in Embase

Interface: **embase.com** content coverage from 1947<br>Date of Search: September 26, 2025<br>Number of hits: 3166<br>Comment: Emtree is the controlled vocabulary in Embase	Field labels<br>• /exp = exploded Emtree term<br>• /de = non exploded Emtree term<br>• ti,ab,kw = title, abstract and author keywords<br>• NEAR/x = within x words, regardless of order<br>• * = truncation of word for alternate endings<br>• $ = 0-1 letter/number<br>• ? = 1 letter/number

No.	Query	Results
#1	'sea food'/exp	25007
#2	'omega 3 fatty acid'/de	45036
#3	'fish oil'/de	21683
#4	'unsaturated fatty acid'/de	18528
#5	'fat intake'/de	58121
#6	'lipid diet'/de	78455
#7	'low fat diet'/de	13568
#8	((algal OR 'cod liver' OR fish OR krill OR marine OR tuna) NEAR/3 oil$):ti,ab,kw	22025
#9	('fatty acid*' NEAR/3 (polyunsaturat* OR unsaturat*)):ti,ab,kw	61799
#10	('fatty acid*' NEAR/3 (n3 OR 'n 3')):ti,ab,kw	14859
#11	'bilantin omega':ti,ab,kw OR 'conchol 36':ti,ab,kw OR 'eicosa e':ti,ab,kw OR eicosapen:ti,ab,kw OR epaisdin:ti,ab,kw OR epanova:ti,ab,kw OR omega3:ti,ab,kw OR 'omega 3':ti,ab,kw OR 'omega forte':ti,ab,kw OR sakana:ti,ab,kw	28732
#12	(('fatty acid*' OR fish) NEAR/5 (consum* OR diet* OR feed* OR intake OR ingest*)):ti,ab,kw	55719
#13	ala:ti,ab,kw OR dha:ti,ab,kw OR epa:ti,ab,kw OR pufa:ti,ab,kw	119090
#14	'alpha-linolenic acid*':ti,ab,kw OR docosahexaenoate:ti,ab,kw OR 'docosahexaenoic acid*':ti,ab,kw OR 'eicosapentaenoic acid*':ti,ab,kw OR icosapent:ti,ab,kw OR linolenate:ti,ab,kw OR 'timnodonic acid*':ti,ab,kw	30689
#15	'fish* product*':ti,ab,kw OR seafood$:ti,ab,kw OR sea-food$:ti,ab,kw OR shellfish*:ti,ab,kw	25302
#16	((diet* OR food* OR high* OR low* OR nutri*) NEAR/5 (fat$ OR fatty)):ti,ab,kw	234705
#17	(fat$ NEAR/2 (alimentary OR consum* OR feed* OR ingest* OR intake)):ti,ab,kw	25335
#18	#1 OR #2 OR #3 OR #4 OR #5 OR #6 OR #7 OR #8 OR #9 OR #10 OR #11 OR #12 OR #13 OR #14 OR #15 OR #16 OR #17	507394

#19	'diet'/de	297460
#20	'dietary pattern'/de	13220
#21	diet$:ti,kw OR dietary:ti,kw OR food$:ti,kw	573910
#22	((diet* OR food*) NEAR/3 (component* OR consum* OR expos* OR factor* OR group* OR ingest* OR intake* OR pattern* OR qualit*)):ab	361436
#23	#19 OR #20 OR #21 OR #22	922917
#24	'risk'/de	507285
#25	'risk factor'/de	1589128
#26	risk*:ti,ab,kw	5084697
#27	#24 OR #25 OR #26	5528429
#28	#23 AND #27	188961
#29	#18 OR #28	656214
#30	'rheumatoid arthritis'/exp/mj	175560
#31	'arthritis'/mj	35846
#32	'rheumatic disease'/mj	41378
#33	'autoimmune disease'/mj	42362
#34	'rheumatoid factor'/mj	5445
#35	'cyclic citrullinated peptide antibody'/de	11739
#36	acpa$:ti,ab,kw OR 'anti ccp':ti,ab,kw OR arthritis:ti,ab,kw OR arthritides:ti,ab,kw OR 'arthrosis deformans':ti,ab,kw OR 'autoimmune disease*':ti,ab,kw OR 'citrullinated peptide antibod*':ti,ab,kw OR 'citrullinated peptide autoantibod*':ti,ab,kw OR 'citrullinated protein autoantibod*':ti,ab,kw OR 'citrullinated protein antibod*':ti,ab,kw OR oligoarthritis:ti,ab,kw OR oligoarthritides:ti,ab,kw OR polyarthritis:ti,ab,kw OR polyarthritides:ti,ab,kw OR rheumatism:ti,ab,kw OR rheumarthritis:ti,ab,kw OR 'rheumatic disease*':ti,ab,kw OR 'rheumatoid factor*':ti,ab,kw	525001
#37	#30 OR #31 OR #32 OR #33 OR #34 OR #35 OR #36	585906
#38	#29 AND #37	5304
#39	#38 NOT ([animals]/lim NOT [humans]/lim)	4558
#40	#39 AND 'Conference Abstract'/it	1392
#41	#39 NOT #40	3166

Supplementary Table 3. Search strategy for identification of relevant articles in Cochrane Library

Interface: **Wiley** content coverage: - Cochrane Database of Systematic Reviews - April 1996<br>Central Trials - Current content July 1998<br>Date of Search: September 26, 2025<br>Number of hits: 696	Field labels<br>• mh = exploded MeSH term<br>• mh ^= non exploded MeSH term<br>• ti,ab,kw = title, abstract and author keywords<br>• NEAR/x = within x words, regardless of order<br>• NEXT = used for truncated phrases<br>• NEXT/x = fixed word order<br>• * = truncation of word for alternate endings<br>• ? = 0-1 letter/number

ID	Search	Hits
#1	[mh Seafood]	264
#2	[mh "Fatty Acids, Omega-3"]	4205
#3	[mh "Fish Oils"]	4648
#4	[mh ^"Fatty Acids, Unsaturated"]	883
#5	[mh "Dietary Fats"]	9687
#6	[mh ^"Diet, High-Fat"]	384
#7	[mh ^"Diet, Fat-Restricted"]	1202
#8	((algal:ti,ab,kw OR "cod liver":ti,ab,kw OR fish:ti,ab,kw OR krill:ti,ab,kw OR marine:ti,ab,kw OR tuna:ti,ab,kw) NEAR/3 oil?:ti,ab,kw)	4043
#9	(("fatty" NEXT acid*):ti,ab,kw NEAR/3 (polyunsaturat*:ti,ab,kw OR unsaturat*:ti,ab,kw))	4547
#10	(("fatty" NEXT acid*):ti,ab,kw NEAR/3 (n3 OR n-3:ti,ab,kw))	2227
#11	("bilantin omega":ti,ab,kw OR "conchol 36":ti,ab,kw OR "eicosa e":ti,ab,kw OR eicosapen:ti,ab,kw OR epaisdin:ti,ab,kw OR epanova:ti,ab,kw OR omega3:ti,ab,kw OR omega-3:ti,ab,kw OR "omega forte":ti,ab,kw OR sakana:ti,ab,kw)	7402
#12	((("fatty" NEXT acid*):ti,ab,kw OR fish:ti,ab,kw) NEAR/5 (consum*:ti,ab,kw OR diet*:ti,ab,kw OR feed*:ti,ab,kw OR intake:ti,ab,kw OR ingest*:ti,ab,kw))	4920
#13	(ala:ti,ab,kw OR dha:ti,ab,kw OR epa:ti,ab,kw OR pufa:ti,ab,kw)	7841
#14	(("alpha-linolenic" NEXT acid*):ti,ab,kw OR docosahexaenoate:ti,ab,kw OR ("docosahexaenoic" NEXT acid*):ti,ab,kw OR ("eicosapentaenoic" NEXT acid*):ti,ab,kw OR icosapent:ti,ab,kw OR linolenate:ti,ab,kw OR ("timnodonic" NEXT acid*):ti,ab,kw)	4714
#15	((fish* NEXT product*):ti,ab,kw OR seafood?:ti,ab,kw OR sea-food?:ti,ab,kw OR shellfish*:ti,ab,kw)	675
#16	((diet*:ti,ab,kw OR food*:ti,ab,kw OR high*:ti,ab,kw OR low*:ti,ab,kw OR nutri*:ti,ab,kw) NEAR/5 (fat?:ti,ab,kw OR fatty:ti,ab,kw))	22679
#17	(fat?:ti,ab,kw NEAR/2 (alimentary:ti,ab,kw OR consum*:ti,ab,kw OR feed*:ti,ab,kw OR ingest*:ti,ab,kw OR intake:ti,ab,kw))	4337
#18	#1 OR #2 OR #3 OR #4 OR #5 OR #6 OR #7 OR #8 OR #9 OR #10 OR #11 OR #12 OR #13 OR #14 OR #15 OR #16 OR #17	37234
#19	[mh ^Diet]	9912
#20	[mh ^"Dietary Patterns"]	0
#21	(diet?:ti,kw OR dietary:ti,kw OR food?:ti,kw)	103426
#22	((diet*:ab OR food*:ab) NEAR/3 (component*:ab OR consum*:ab OR expos*:ab OR factor*:ab OR group*:ab OR ingest*:ab OR intake*:ab OR pattern*:ab OR qualit*:ab))	38815
#23	#19 OR #20 OR #21 OR #22	116942
#24	[mh ^Risk]	4529
#25	[mh ^"Risk Factors"]	37615
#26	risk*:ti,ab,kw	336663
#27	#24 OR #25 OR #26	336663

#28	#23 AND #27	26512
#29	#18 OR #28	57440
#30	[mh "Arthritis, Rheumatoid"]	8188
#31	[mh ^Arthritis]	2521
#32	[mh ^"Rheumatic Diseases"]	485
#33	[mh ^"Autoimmune Diseases"]	448
#34	[mh ^"Rheumatoid Factor"]	165
#35	[mh ^"Anti-Citrullinated Protein Antibodies"]	28
#36	(ACPA?:ti,ab,kw OR "anti CCP":ti,ab,kw OR arthritis:ti,ab,kw OR arthritides:ti,ab,kw OR "arthrosis deformans":ti,ab,kw OR ("autoimmune" NEXT disease*):ti,ab,kw OR ("citrullinated peptide" NEXT antibod*):ti,ab,kw OR ("citrullinated peptide" NEXT autoantibod*):ti,ab,kw OR ("citrullinated protein" NEXT autoantibod*):ti,ab,kw OR ("citrullinated protein" NEXT antibod*):ti,ab,kw OR oligoarthritis:ti,ab,kw OR oligoarthritides:ti,ab,kw OR polyarthritis:ti,ab,kw OR polyarthritides:ti,ab,kw OR rheumatism:ti,ab,kw OR rheumarthritis:ti,ab,kw OR ("rheumatic" NEXT disease*):ti,ab,kw OR ("rheumatoid" NEXT factor*):ti,ab,kw)	34691
#37	#30 OR #31 OR #32 OR #33 OR #34 OR #35 OR #36	35046
#38	#29 AND #37	696

Supplementary Table 4. Studies that were excluded after full text screening and reasons for exclusion

Reason	Number of studies	References
Ineligible study design	7	(1-7)
Ineligible exposure	26	(8-33)
Ineligible outcome	1	(34)
Ineligible publication type	3	(35-37)
Estimates not reported	2	(38, 39)

Supplementary Table 5. Characteristics of studies included in meta-analysis

Study	Country	Study name	Study design	Sample size	Cases	Sex	Age, years	Exposure	Exposure assessment	RA assessment	RR (95% CI)	Adjusted factors
Bäcklund 2025(40)	Sweden	Malmö Diet and Cancer Study	Nested case-control	1525	305	Men and women	41–73	Fish and shellfish: 614.1 g/week vs 64.3 g/week	Modified diet history method	National and local registers	1.47 (0.86, 2.51)	Sex, year of birth, year of inclusion, total energy intake, smoking, alcohol, physical activity
Chen 2025(41)	UK	UK Biobank	Cohort	188597	1640	Men and women	40–69	Dietary omega-3 ALA+SDA+EPA +DPA+ DHA: per 1 SD increase	Oxford WebQ questionnaire (a 24-hour dietary assessment tool)	Inpatient hospital records	0.91 (0.85, 0.98)	Age, sex, race and ethnicity, body mass index, alcohol intake, smoking status, metabolic equivalent task, Townsend deprivation index, energy intake, vitamin A intake, and vitamin E intake
								Dietary omega-3 ALA: per 1 SD increase			1.00 (0.93, 1.07)	
								Dietary omega-3 SDA: per 1 SD increase			0.91 (0.86, 0.96)	
								Dietary omega-3 EPA: per 1 SD increase			0.90 (0.85, 0.95)	
								Dietary omega-3 DPA: per 1 SD increase			0.91 (0.86, 0.96)	
								Dietary omega-3 DHA: per 1 SD increase			0.90 (0.85, 0.95)	
				44445	1640			Plasma omega-3 ALA+SDA+EPA +DPA+ DHA: per 1 SD increase	High-throughput nuclear magnetic resonance (NMR)–based metabolic biomarker profiling platform		0.83 (0.75, 0.93)	
								Plasma omega-3 DHA: per 1 SD increase			0.83 (0.74, 0.93)	
de Pablo 2018(42)	Italy & Spain	EPIC	Nested case-control	354	96	Men and women	30–70	Erythrocyte omega-3: 7.33% vs 4.89% of total	Peak area using HP Chem	Linkage with healthcare records,	1.64 (0.77, 3.52)	Age, sex, centre, date and time of baseline blood collection, fasting status at

Study	Country	Study name	Study design	Sample size	Cases	Sex	Age, years	Exposure	Exposure assessment	RA assessment	RR (95% CI)	Adjusted factors
								erythrocyte membrane fatty acids	Station	hospital and drug prescription databases, validated through medical record review		blood collection, body mass index, waist circumference, education level, physical activity, smoking status and alcohol intake
								Erythrocyte omega-3 ALA: 0.48% vs 0.10%			1.02 (0.52, 1.99)	
								Erythrocyte omega-3 ETA: 0.08% vs 0.03%			1.82 (0.86, 3.83)	
								Erythrocyte omega-3 EPA: 1.07% vs 0.43%			1.26 (0.55, 2.88)	
								Erythrocyte omega-3 DPA: 0.015% vs 0.010%			1.70 (0.84, 3.45)	
								Erythrocyte omega-3 DHA: 1.97% vs 1.58%			1.54 (0.73, 3.26)	
Di Giuseppe 2014(43)	Sweden	Swedish Mammography Cohort	Cohort	32232	205	Women	54–89	Fish: ≥1 vs < 1 serving/week	FFQ	Swedish Rheumatology Register (SRR) and the Outpatient Register of the Swedish National Board of Health and Welfare	0.71 (0.48, 1.04)	Age, cigarette smoking, alcohol intake, use of aspirin, red meat consumption, dairy food consumption, and energy intake
								Dietary omega-3 EPA+ DPA + DHA: 0.62 vs 0.15 g/day			0.67 (0.44, 1.03)	Age, cigarette smoking, alcohol intake, use of aspirin and energy intake
Fang 2023(44)	UK	UK Biobank	Cohort	93800	620	Men and women	40–69	Plasma omega-3: per 1 SD increase	High-throughput nuclear magnetic resonance (NMR)–based metabolic biomarker profiling platform	Inpatient hospital records	0.88 (0.80, 0.96)	Age, sex, assessment centre, fasting time, education level, smoking status, body mass index, and physical activity duration
								% of omega-3 in total fatty acids: per 1 SD increase			0.86 (0.79, 0.94)	
								Plasma omega-3 DHA: per 1 SD increase			0.87 (0.79, 0.95)	
								% of omega-3 DHA in total fatty			0.88 (0.81, 0.97)	

Study	Country	Study name	Study design	Sample size	Cases	Sex	Age, years	Exposure	Exposure assessment	RA assessment	RR (95% CI)	Adjusted factors
								acids: per 1 SD increase				
Gan 2017a(45)	US	SERA only anti-CCP+	Cohort	35	14	Men and women	≥18	Erythrocyte omega-3: per 1 SD increase in % of total erythrocyte membrane fatty acids	Gas chromatography with mass spectral detection	Rheumatologist diagnosis	0.51 (0.22, 1.19)	Age, SE+, RF+, CRP+ and omega-3 supplement use
								Erythrocyte omega-3 EPA+DHA: per 1 SD increase			0.59 (0.27, 1.26)	
								Erythrocyte omega-3 ALA: per 1 SD increase			2.01 (0.84, 4.81)	
								Erythrocyte omega-3 EPA: per 1 SD increase			0.78 (0.48, 1.28)	
								Erythrocyte omega-3: DPA per 1 SD increase			0.52 (0.27, 0.98)	
								Erythrocyte omega-3 DHA: per 1 SD increase			0.60 (0.26, 1.36)	
Hatami 2022(46)	Iran	NA	Hospital-based case-control	297	100	Men and women	19–69	Fish and sea food: >median vs <median	FFQ assessing consumption 1 year before diagnosis	Rheumatology clinic	0.52 (0.27, 0.98)	Age, sex, body mass index, physical activity, total energy intake, smoking, socioeconomic status, drug use and family disease history
He 2016(47)	China	NA	Hospital-based case-control	2005	968	Men and women	>18	Fish: per kg/month	FFQ assessing consumption 5 years before diagnosis	Hospital diagnosis	0.86 (0.65, 1.16)	Geographic area, gender, age, ethnicity, body mass index, and intakes of red meat, poultry, viscera, potato, vegetables, mushroom, bean, citrus, other fruits, dairy products, eggs

Study	Country	Study name	Study design	Sample size	Cases	Sex	Age, years	Exposure	Exposure assessment	RA assessment	RR (95% CI)	Adjusted factors
Hu 2017(48)	US	NHS and NHS II	Cohort	169989	444	Women	25–55	Dietary omega-3 EPA+ DHA: 250 vs 63 mg/day	FFQ	Self-reported diagnosis confirmed by Connective Tissue Disease Screening Questionnaire and by medical record review	0.86 (0.66, 1.13)	Age, cohort, smoking, age at menarche, parity, breastfeeding, hormone use, total energy, and body mass index
Krok-Schoen 2018(49)	US	Women's Health Initiative	Cohort	80551	3348	Women	55–79	Dietary omega-3 EPA+ DPA + DHA: >203.3 vs <56.2 mg/day	FFQ	Self-report of physician diagnosis	1.01 (0.90, 1.13)	Age, Women's Health Initiative study component, energy, education, race/ethnicity, body mass index, memory loss, and fruit intake
								Dietary omega-3 ALA: >1632.2 vs <916.9 mg/day			1.00 (0.89, 1.12)	
								Dietary omega-3 EPA: >63.2 vs <15.4 mg/day			1.03 (0.92, 1.16)	
								Dietary omega-3 DPA: >21.9 vs <6.7 mg/day			1.00 (0.90, 1.12)	
								Dietary omega-3 DHA: >119.5 vs <32.4 mg/day			0.99 (0.88, 1.11)	
Kronzer 2022(50)	US	Mayo Clinic Biobank	Hospital-based case-control	269	67	Men and women	Mean: 64	Fish: > 2 vs ≤ 1 times/day	Questionnaire assessing consumption 2 years before diagnosis	Electronic health records using a rules-based algorithm combining two diagnosis codes with use of a disease-modifying anti-rheumatic drug	0.52 (0.17, 1.61)	Age, sex, race/ethnicity, education, smoking status, asthma, parity, recruitment year, recruitment location, and distance from recruitment location
				848	212			Any vs no fish oil/omega fatty acid supplements 2 times/week for at least 3 months			1.08 (0.76, 1.56)	
Linos 1991(51)	Greece	NA	Hospital-based case-control	305	168	Men and women	24–89	Fish: ≥2 vs <2 times/week	Dietary questionnaire covering the entire	Two hospitals	0.66 (0.25, 1.75)	Age, sex, occupation, residence, marital status, body mass index, adherence to Orthodox Lent, olive oil

Study	Country	Study name	Study design	Sample size	Cases	Sex	Age, years	Exposure	Exposure assessment	RA assessment	RR (95% CI)	Adjusted factors
									life before diagnosis			consumption, other dietary items
Linos 1999(52)	Greece	NA	Hospital-based case-control	333	145	Men and women	18–80	Fish: 10 vs 3 servings/month	Dietary questionnaire covering the entire life before diagnosis	Two hospitals and one outpatient clinic	0.95 (0.46, 1.96)	Age, sex, healthcare facility
Mazzucca 2022(53)	UK	UK biobank	Cohort	479494	2819	Men and women	40–69	Oily fish: ≥2 vs 0 times/week	FFQ	Inpatient hospital records	0.86 (0.75, 0.99)	Age, sex, study area, income, smoking, body mass index, physical activity, comorbidities, alcohol consumption.
Nguyen 2022a(54)	France	E3N Study	Cohort	62629	480	Women	40–65	Fish: 31.1–261 vs 0–16.7 g/day	FFQ	Algorithm based on repeated self-report, confirmation via a RA-specific questionnaire and clinical criteria, or use of RA-specific medications	0.99 (0.80, 1.22)	Age, total daily intake, body mass index, cigarette smoking, exposure to passive smoking in childhood, gastrointestinal transit, educational level, and physical activity
								Oily fish: 14.3-261 vs 0-5.7 g/day			1.01 (0.81, 1.25)	
Pedersen 2005(55)	Denmark	Diet, Cancer, and Health cohort	Cohort	57053	69	Men and women	50–64	Fish: per 30 g/day	FFQ	Danish National Patient Registry	0.91 (0.68, 1.23)	Age and gender
								Oily fish: per 30 g/day			0.51 (0.25, 1.03)	Age, gender, tobacco smoking, education, and medium fat fish
Rosell 2009(56)	Sweden	EIRA	Population-based case-control	4034	1889	Men and women	18–70	Oily fish: 1-7 times/week vs never or seldom	Questionnaire assessing consumption 5 years before diagnosis	All public rheumatology units in the study area	0.80 (0.60, 1.00)	Age, residential area, smoking, and sex
								Any fish oil supplements vs no omega-3 supplements			1.10 (0.80, 1.60)	
Shapiro 1996(57)	US	King county	Population-based	1569	324	Women	15–64	Fish and shellfish: ≥2 vs <1 servings/week	FFQ assessing consumption	Surveillance of rheumatologists in the study	0.92 (0.67, 1.25)	Age, reference year, education, race, total caloric intake

Study	Country	Study name	Study design	Sample size	Cases	Sex	Age, years	Exposure	Exposure assessment	RA assessment	RR (95% CI)	Adjusted factors
		residents and GHC	case-control					Dietary omega-3 (long chain): >1.6 vs ≤0.2 g/day	5 years before diagnosis	area and medical record review	0.77 (0.46, 1.27)	
Sparks 2019(58)	US	NHS and NHS II	Cohort	166013	1080	Women	25–55	Fish: ≥4 vs 0 to <1 serving/week	FFQ	Self-reported diagnosis confirmed by Connective Tissue Disease Screening Questionnaire and by medical record review	0.93 (0.67, 1.28)	Age, questionnaire period, cohort, total energy intake, median household income, cigarette smoking, body mass index, and alcohol intake
								Dietary and supplementary omega-3 EPA+DHA+DPA: Q4 vs Q1			1.12 (0.91, 1.37)	Age, questionnaire period, cohort, total energy intake, median household income, cigarette smoking, body mass index, alcohol intake, ALA, trans fatty acids, saturated fatty acids, monounsaturated fatty acids, polyunsaturated fatty acids, and protein
Sundström 2015(59)	Sweden	VIP	Nested case-control	2272	386	Men and women	30–60	Fish: Q3 vs Q1	FFQ	Hospital medical records	0.94 (0.71, 1.26)	Age, sex, date of examination, version of the FFQ, smoking status and education
								Dietary omega-3 (long chain): Q3 vs Q1			1.04 (0.80, 1.37)	

ALA, alpha-linolenic acid (18:3); anti-CCP, anti-cyclic citrullinated peptide; CI, confidence interval; CRP, C-reactive protein; DHA, docosahexaenoic acid (22:6); DPA, docosapentaenoic acid (22:5); EIRA, Epidemiological Investigation of Rheumatoid Arthritis; EPA, eicosapentaenoic acid (20:5); EPIC, European Prospective Investigation into Cancer and Nutrition; ETA, eicosatetraenoic acid (20:4); FFQ, food frequency questionnaire; GHC, Group Health Cooperative; NHS, Nurses' Health Study; RA, rheumatoid arthritis; RF, rheumatoid factor; RR, risk ratio; SD, standard deviation; SDA, stearidonic acid (18:4); SE, shared epitope; SERA, Studies of the Etiology of Rheumatoid Arthritis; VIP, Västerbotten Intervention Program

Supplementary Table 6. Characteristics of eligible studies not included in meta-analysis

Study	Country	Study name	Study design	Sample size	Cases	Sex	Age, years	Exposure	Exposure assessment	RA assessment	RR (95% CI)	Adjusted factors
Cross-sectional												
Kang 2024(60)	US	NHANES 1999-2020	Cross-sectional	50352	3007	Men and women	≥18	Dietary omega-3 DHA + DPA+ EPA+SDA + ALA: ≥ 24.89 vs ≤ 13.53 mg/kg/day	24-hour dietary recall	Self-report of physician diagnosis	0.72 (0.60, 0.86)	Age, gender, race, educational level, income, marital status, drinking status, smoking status, and total energy intake
Lee 2013(61)	Korea	NA	Cross-sectional	200	100	Women	18-80	Erythrocyte omega-3 ALA+EPA+DHA : >13.94% vs ≤ 12.03%	Gas chromatography	Five hospitals	1.06 (0.53, 2.11)	Age, sex, body weight, and smoking status
								Erythrocyte omega-3 EPA+DHA: >11.32% vs ≤ 9.74%			0.59 (0.29, 1.19)	
								Erythrocyte omega-3 ALA: >0.83% vs ≤ 0.46%			0.03 (0.01, 0.12)	
								Erythrocyte omega-3 EPA: >2.58% vs ≤ 1.90%			0.38 (0.18, 0.79)	
								Erythrocyte omega-3 DHA: >8.70% vs ≤ 7.35%			1.00 (0.50, 2.03)	
Nguyen 2022b(62)	Korea	KNHANES 2009-2019	Cross-sectional	33966	1078	Men and women	>50	Dietary omega-3: per 2-fold increase in daily intake	24-hour dietary recall	Self-report of physician diagnosis	0.92 (0.83, 1.02)	Age, sex, energy intake, residential area, monthly household income, education levels, physical activity, body mass index group, high risk drinking, occupation, and smoking

Study	Country	Study name	Study design	Sample size	Cases	Sex	Age, years	Exposure	Exposure assessment	RA assessment	RR (95% CI)	Adjusted factors
												status, cotinine verified smoker, comorbidities
Nguyen 2022c(63)	Korea	KNHANES 2009-2019	Cross-sectional	56462	1187	Men and women	18-80	Dietary omega-3: per 2-fold increase in daily intake	24-hour dietary recall	Self-report of physician diagnosis	0.98 (0.93, 1.04)	Age, sex, energy intake, monthly household income, education levels, physical activity, body mass index group, high risk drinking, occupation, and smoking status, cotinine verified smoker
Tu 2025(64)	China	NA	Cross-sectional	432	216	Men and women	≥18	Fish: 5-7 vs < 3 times/week	Questionnaire	Two hospitals	7.96 (1.89, 33.55)	Sex, age, occupation type, place of residence, first-degree relatives, second-degree relatives, chronic disease status, periodontal disease status, history of tuberculosis, history of mumps, allergy history, dietary combination, breakfast habits, taste type, animal viscera, regular meals, dairy products, sweet, drinking alcohol, damp dwelling, living above the second floor, dust exposure, and vaccine history, rs805297, rs9272219, HLA0405
Xie 2023(65)	US	NHANES 2010-2020	Cross-sectional	16530	1053	Men and women	≥18	Dietary omega-3 DHA: Q4 vs ≤ Q1	Two 24-hour dietary recalls	Self-report of physician diagnosis	0.69 (0.53, 0.90)	Sex, age, race, education, family income-to-poverty ratio, body mass index, diabetes, energy, protein, carbohydrate, fat, sugar, fiber, alcohol and caffeine
								Dietary omega-3 DPA: Q4 vs ≤ Q1			1.18 (0.91, 1.53)	

Study	Country	Study name	Study design	Sample size	Cases	Sex	Age, years	Exposure	Exposure assessment	RA assessment	RR (95% CI)	Adjusted factors
								Dietary omega-3 EPA: Q4 vs ≤ Q1			0.88 (0.68, 1.14)	
								Dietary omega-3 SDA: Q4 vs ≤ Q1			0.91 (0.70, 1.18)	
								Dietary omega-3 ALA: Q4 vs ≤ Q1			0.99 (0.76, 1.29)	
Overlapping data with other cohorts												
Bäcklund 2024(66)	Sweden	Malmö Diet and Cancer Study	Nested case-control	860	172	Men and women	41–73	Fish and shellfish: per 1 SD increase	Modified diet history method	National and local registers	1.16 (0.90, 1,49)	Sex, year of birth, year of inclusion, total energy intake, smoking, alcohol, physical activity
Benito-Garcia 2007(67)	US	NHS	Cohort	82063	546	Women	30–55	Fish: 0.44 vs 0.07 servings/day	FFQ	Self-reported diagnosis confirmed by Connective Tissue Disease Screening Questionnaire and by medical record review	0.96 (0.72, 1,26)	Age, total energy intake, body mass index, smoking, and total lifetime breastfeeding
Hu 2015(68)	US	NHS and NHS II	Cohort	174638	913	Women	25–55	Fish: Q4 vs Q1	FFQ	Self-reported diagnosis confirmed by Connective Tissue Disease Screening Questionnaire and by	1.15 (0.95, 1.40)	Age, census tract median family income, cigarette smoking status, age at menarche, parity and breastfeeding, hormone use, physical activity, body mass index, multivitamin use, diabetes mellitus history, sugar-sweetened soda

Study	Country	Study name	Study design	Sample size	Cases	Sex	Age, years	Exposure	Exposure assessment	RA assessment	RR (95% CI)	Adjusted factors
										medical record review		consumption, coffee consumption, and total energy
Nguyen 2021(69)	France	E3N Study	Cohort	62629	480	Women	40–65	Fish: >25 vs <9 g/day	FFQ	Algorithm based on repeated self-report, confirmation via a RA-specific questionnaire and clinical criteria, or use of RA-specific medications	0.99 (0.80, 1.22)	Age, total daily intake, body mass index, cigarette smoking, exposure to passive smoking in childhood, gastrointestinal transit, educational level, and physical activity
VITAL trial												
Costenbader 2024(70)	US	VITAL	RCT	25871	45	Men and women	≥50	Omega-3 supplement (1 g/day as a fish oil capsule containing 460 mg EPA and 380 mg of DHA) vs placebo for 5 years	NA	Self-report of physician diagnosis during the randomized time, complemented by review of medical records through 2022	0.66 (0.37, 1.21)	Age, sex, race, and vitamin D randomization group
					64					Self-report of physician diagnosis during the randomized time with a 2-year observational extension, complemented by review of medical	0.64 (0.39, 1.06)	

Study	Country	Study name	Study design	Sample size	Cases	Sex	Age, years	Exposure	Exposure assessment	RA assessment	RR (95% CI)	Adjusted factors
										records through 2022		
Hahn 2022(71)	US	VITAL	RCT	25871	39	Men and women	≥50	Omega-3 supplement (1 g/day as a fish oil capsule containing 460 mg EPA and 380 mg of DHA) vs placebo for 5 years	NA	Self-report of physician diagnosis during the randomized time, complemented by review of medical records through 2019	0.58 (0.30, 1.13)	Age, sex, race, and vitamin D randomization group
Preclinical RA												
Gan 2016(72)	US	SERA	Nested case-control	77	30	Men and women	≥18	Any vs no fish oil, fish liver oil or omega-3 or use of multivitamins that contained omega-3 in the past year	Questionnaire	Anti-CCP2 positive on at least one visit, and never testing anti-CCP2 negative at subsequent visits	0.11 (0.02, 0.61)	Age at visit, sex, race, site, current smoker, shared epitope, education and income
								Erythrocyte omega-3: per 1 SD increase in % of total erythrocyte membrane fatty acids	Gas chromatography with mass spectral detection		0.44 (0.21, 0.92)	
								Erythrocyte omega-3 EPA+DHA: per 1 SD increase			0.47 (0.23, 0.97)	
								Erythrocyte omega-3 ALA: per 1 SD increase			0.87 (0.52, 1.45)	
								Erythrocyte omega-3 EPA: per 1 SD increase			0.55 (0.25, 1.21)	
								Erythrocyte omega-3: DPA per 1 SD increase			0.65 (0.38, 1.13)	

Study	Country	Study name	Study design	Sample size	Cases	Sex	Age, years	Exposure	Exposure assessment	RA assessment	RR (95% CI)	Adjusted factors
								Erythrocyte omega-3 DHA: per 1 SD increase			0.50 (0.26, 0.97)	
Gan 2017b(73)	US	SERA	Nested case-control	80	11	Men and women	≥18	Erythrocyte omega-3: per 1 SD increase in % of total erythrocyte membrane fatty acids	Gas chromatography with mass spectral detection	Anti-CCP2 and SE positive	0.26 (0.09, 0.77)	Age at visit, sex, race/ethnicity, site, current smoker, income and education
				85	16					Anti-CCP2 positive and SE negative	0.92 (0.48, 1.77)	
				93	24					High titre RF by nephelometry, anti-CCP2 negative, and SE positive	0.44 (0.21, 0.93)	
				85	16					High titre RF by nephelometry, anti-CCP2 negative, and SE negative	0.77 (0.41, 1.45)	

ALA, alpha-linolenic acid (18:3); anti-CCP, anti-cyclic citrullinated peptide; CI, confidence interval; DHA, docosahexaenoic acid (22:6); DPA, docosapentaenoic acid (22:5); EPA, eicosapentaenoic acid (20:5); ETA, eicosatetraenoic acid (20:4); FFQ, food frequency questionnaire; KNHANES, Korean National Health and Nutrition Examination Survey; NHANES, National Health and Nutrition Examination Survey; NHS, Nurses' Health Study; RA, rheumatoid arthritis; RCT, randomized controlled trial; RF, rheumatoid factor; RR, risk ratio; SD, standard deviation; SDA, stearidonic acid (18:4); SE, shared epitope; SERA, Studies of the Etiology of Rheumatoid Arthritis; VITAL, vitamin D and omega-3 trial

Supplementary Table 7. Certainty of evidence assessment using the GRADE system

Certainty assessment							N	Cases	Comparison	Pooled risk ratio (95% CI)	Certainty	Outcome
Studies	Study design	Risk of bias	Inconsistency	Indirectness	Imprecision	Other considerations						
**Fish consumption**												
12	6 Cohorts<br>6 Case-control	serious[1]	not serious	not serious	not serious	Non-linear dose-response relationship<br>Possibility of publication bias<br>RR 0.90 (0.81 to 1.00) after excluding studies at high risk of bias	269618	4132	High vs low	**RR 0.88**<br>(0.81 to 0.96)	⊕⊕⊕◯<br>MODERATE	RA
12	7 Cohorts<br>5 Case-control	serious[1]	not serious	not serious	not serious	RR 1.01 (0.99 to 1.03) after excluding studies at high risk of bias	809464	8838	Per serving/week	**RR 1.00**<br>(0.98 to 1.02)	⊕⊕⊕◯<br>MODERATE	RA
**Dietary omega-3 fatty acids**												
5	4 Cohorts<br>1 Case-control	serious[2]	not serious	not serious	serious[3]	RR 0.80 (0.64 to 1.01) after excluding studies at high risk of bias	132707	2138	High vs low	**RR 0.94**<br>(0.83 to 1.07)	⊕⊕◯◯<br>LOW	RA
6	5 Cohorts<br>1 Case-control	serious[2]	not serious	not serious	not serious	RR 0.73 (0.44 to 1.21) after excluding studies at high risk of bias	475210	6347	Per 1 g/day	**RR 0.91**<br>(0.85 to 0.97)	⊕⊕⊕◯<br>MODERATE	RA
**Omega-3 fatty acid supplementation**												

Certainty assessment							N	Cases	Comparison	Pooled risk ratio (95% CI)	Certainty	Outcome
Studies	Study design	Risk of bias	Inconsistency	Indirectness	Imprecision	Other considerations						
2	Case-control	very serious[4]	not serious	not serious	very serious[5]	None	4513	342	Any vs none	**RR 1.09** (0.85 to 1.40)	⊕◯◯◯ VERY LOW	RA
**Circulating omega-3 fatty acids**												
3	Cohorts	serious[6]	serious[7]	serious[8]	very serious[5]	Smoking explained heterogeneity<br><br>RR 1.01 (0.76 to 1.33) after excluding study at high risk of bias	94189	730	Per 1% increase	**RR 0.94** (0.74 to 1.18)	⊕◯◯◯ VERY LOW	RA

CI, confidence interval; RA, rheumatoid arthritis; RR, risk ratio

[1]At least half of the studies were considered to have a high risk of bias, mainly due to potential differential exposure misclassification in case-control studies. However, excluding studies at high risk of bias yielded a similar estimate. Therefore, we decided to downgrade by one level.

[2]At least half of the studies were considered to have a high risk of bias, mainly due to post-exposure interventions. However, excluding studies at high risk of bias resulted in a larger effect size. Therefore, we decided to downgrade by one level.

[3]The confidence interval includes the possibility of both harm (or no effect) and benefit, but it is not considerably wide. Therefore, we decided to downgrade by one level.

[4]All studies were considered to have a high risk of bias, mainly due to potential differential exposure misclassification. Therefore, we decided to downgrade by two levels.

[5]The confidence interval includes the possibility of both harm (or no effect) and benefit, and it is considerably wide. Therefore, we decided to downgrade by two levels.

[6]One study was considered to have a high risk of bias due to insufficient confounding adjustment. Excluding this study attenuated the estimate. Therefore, we decided to downgrade by one level.

[7]There was moderate heterogeneity ($I^2 > 50\%$), and not all studies pointed towards benefit. Therefore, we decided to downgrade by one level.

[8]There were differences in biological compartments used to assess omega-3 status (plasma vs erythrocyte fatty acids). Therefore, we decided to downgrade by one level.

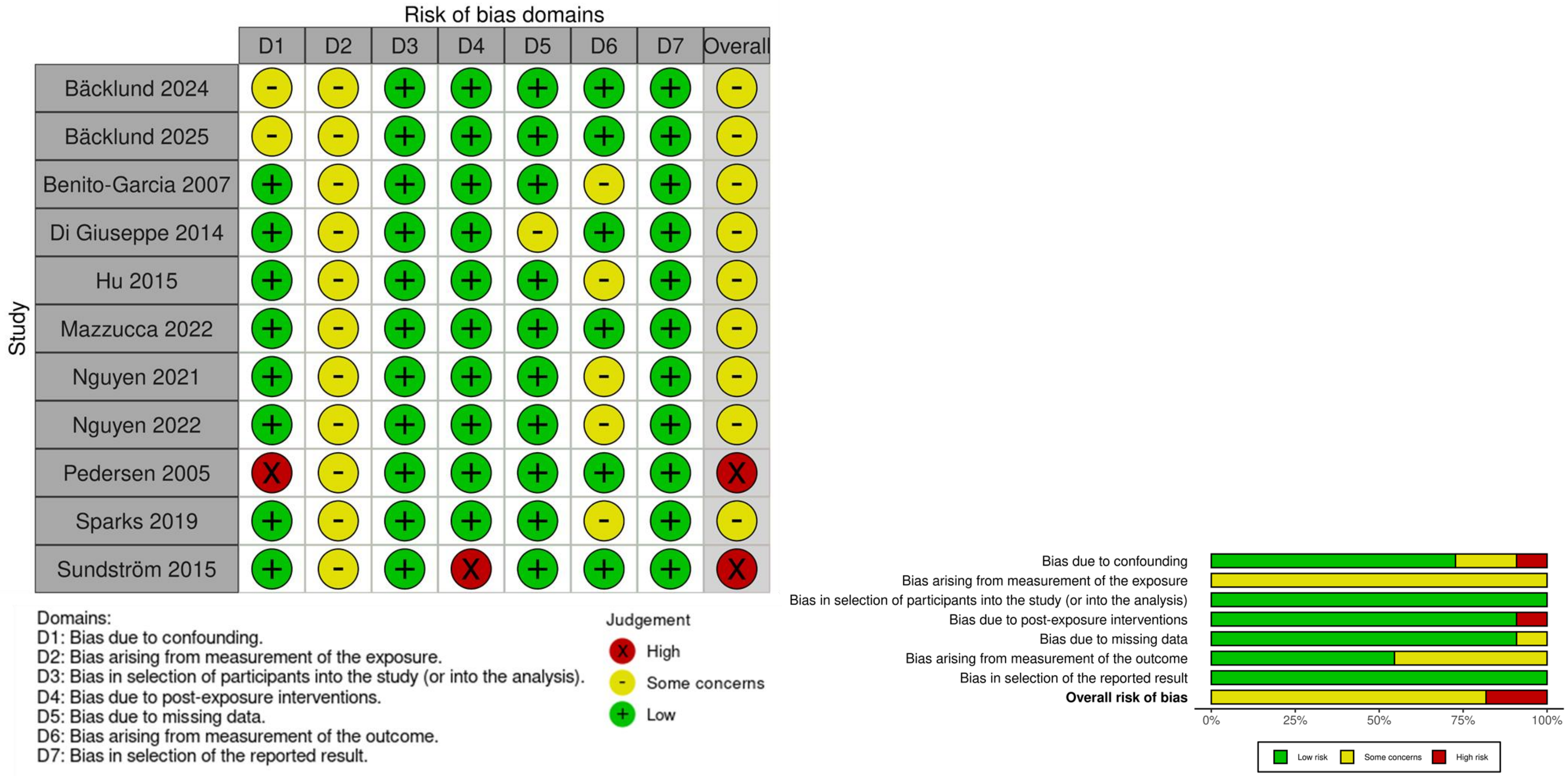

Supplementary Figure 1. Risk of bias assessment of cohort and nested case-control studies on fish consumption using ROBINS-E, generated with the robvis tool (74)

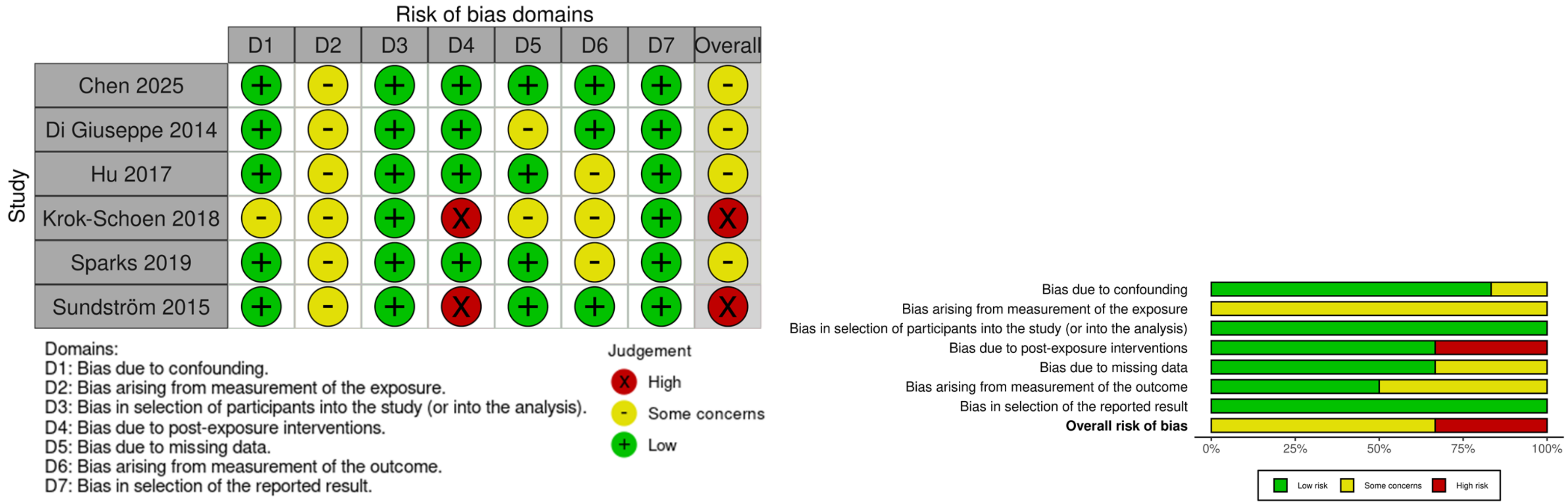

Supplementary Figure 2. Risk of bias assessment of cohort and nested case-control studies on dietary omega-3 fatty acids using ROBINS-E, generated with the robvis tool (74)

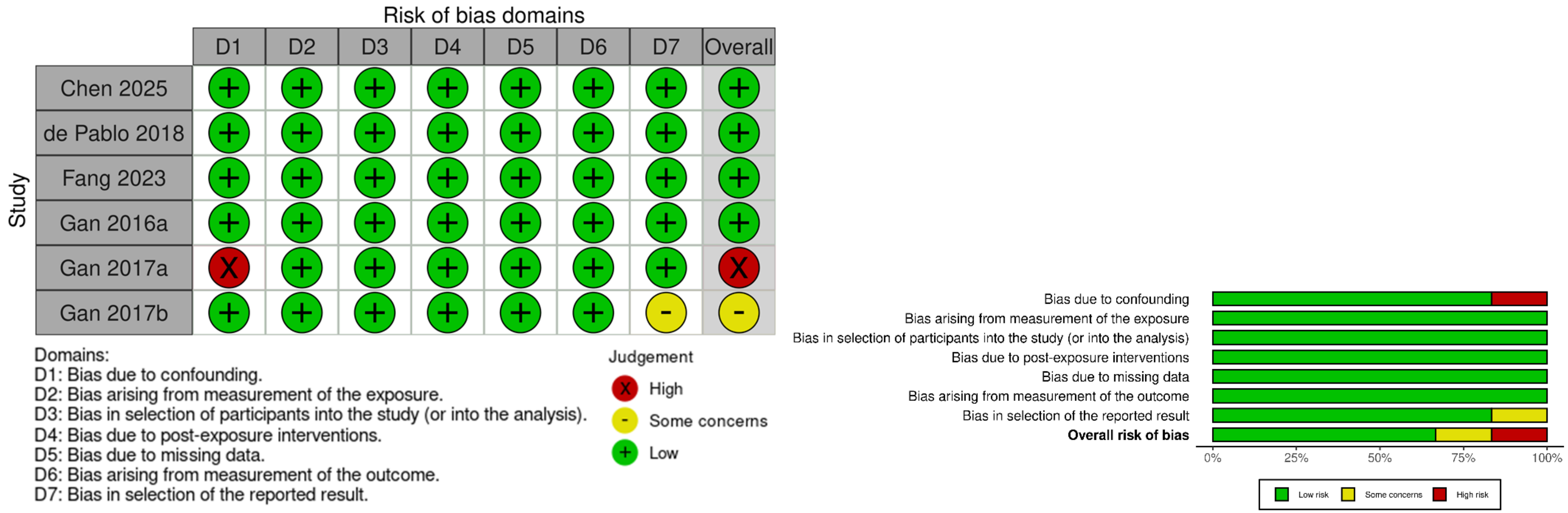

Supplementary Figure 3. Risk of bias assessment of cohort and nested case-control studies on circulating omega-3 fatty acids using ROBINS-E, generated with the robvis tool (74)

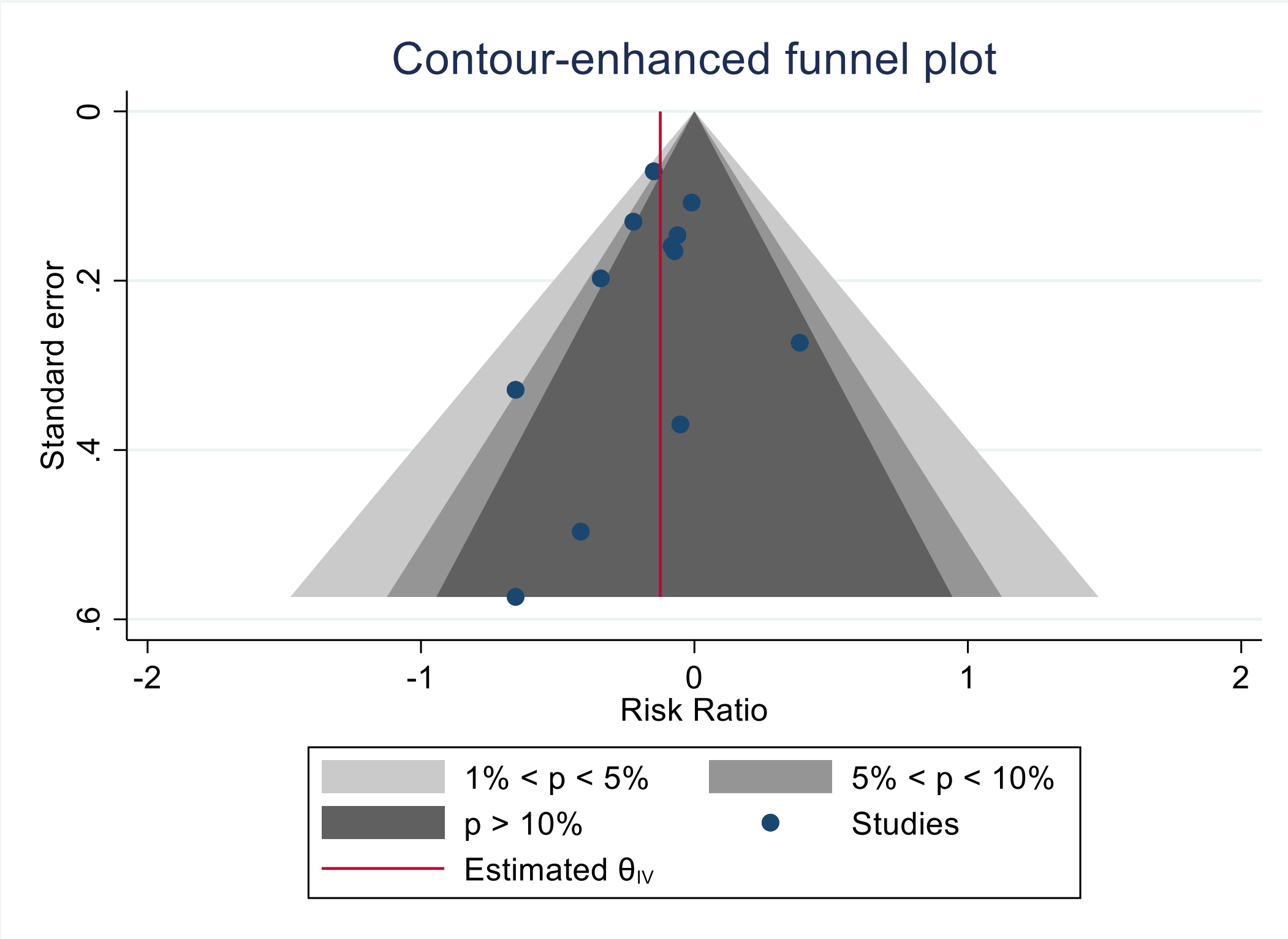

Supplementary Figure 4. Contour-enhanced funnel plot of risk ratios for rheumatoid arthritis comparing high versus low fish consumption. Egger's test *P* = 0.5106.

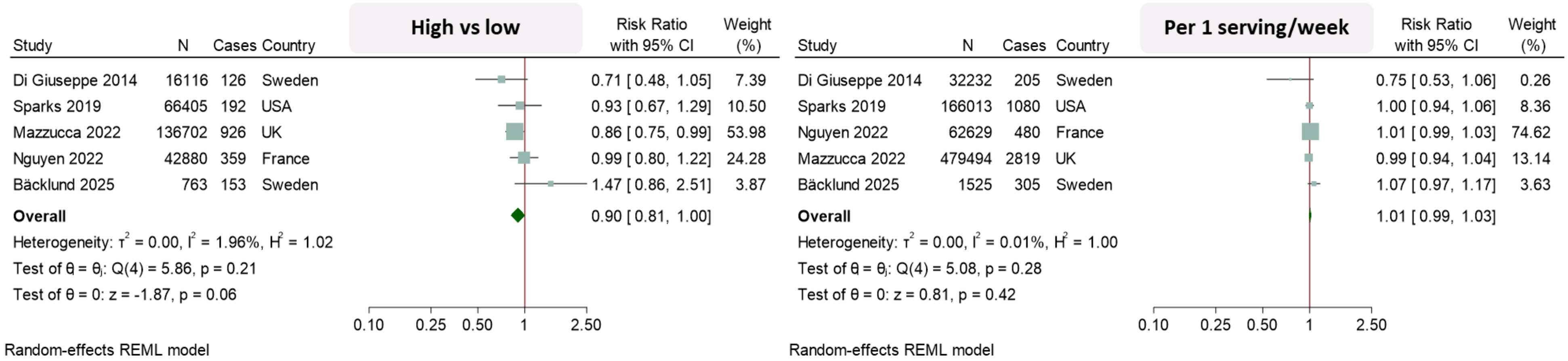

Supplementary Figure 5. Forest plots of risk ratios for rheumatoid arthritis comparing high versus low fish consumption and per 1 serving/week increase, after excluding studies at high risk of bias. CI, confidence interval.

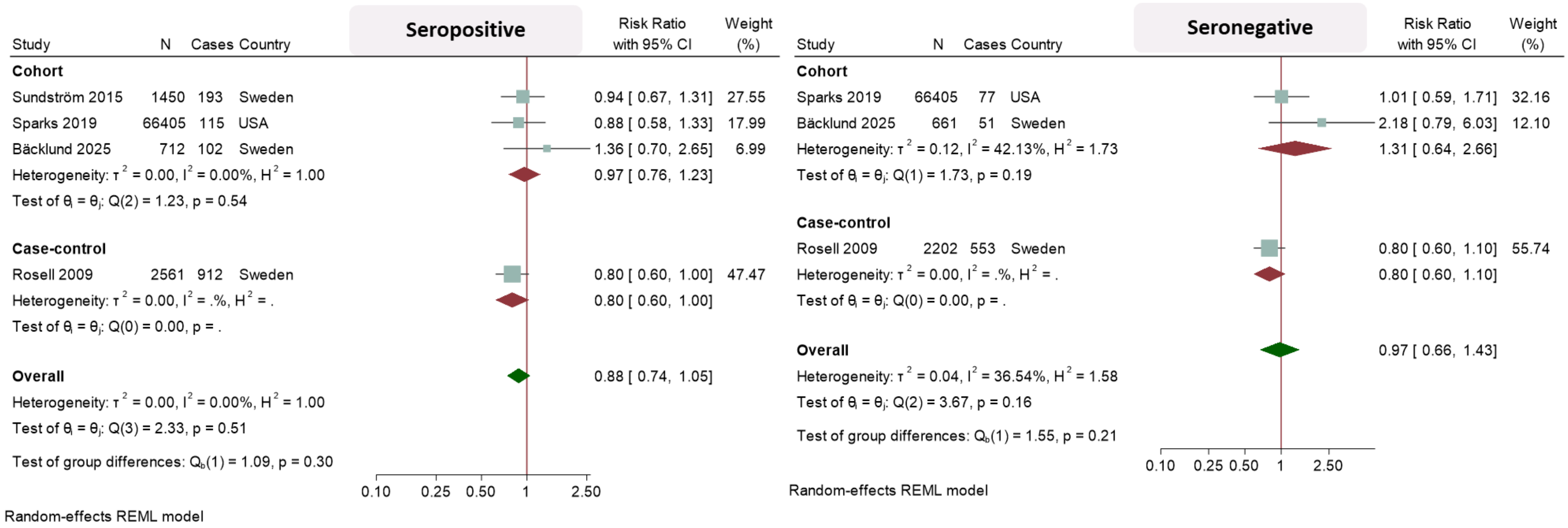

Supplementary Figure 6. Forest plots of risk ratios for seropositive and seronegative rheumatoid arthritis comparing high versus low fish consumption. CI, confidence interval.

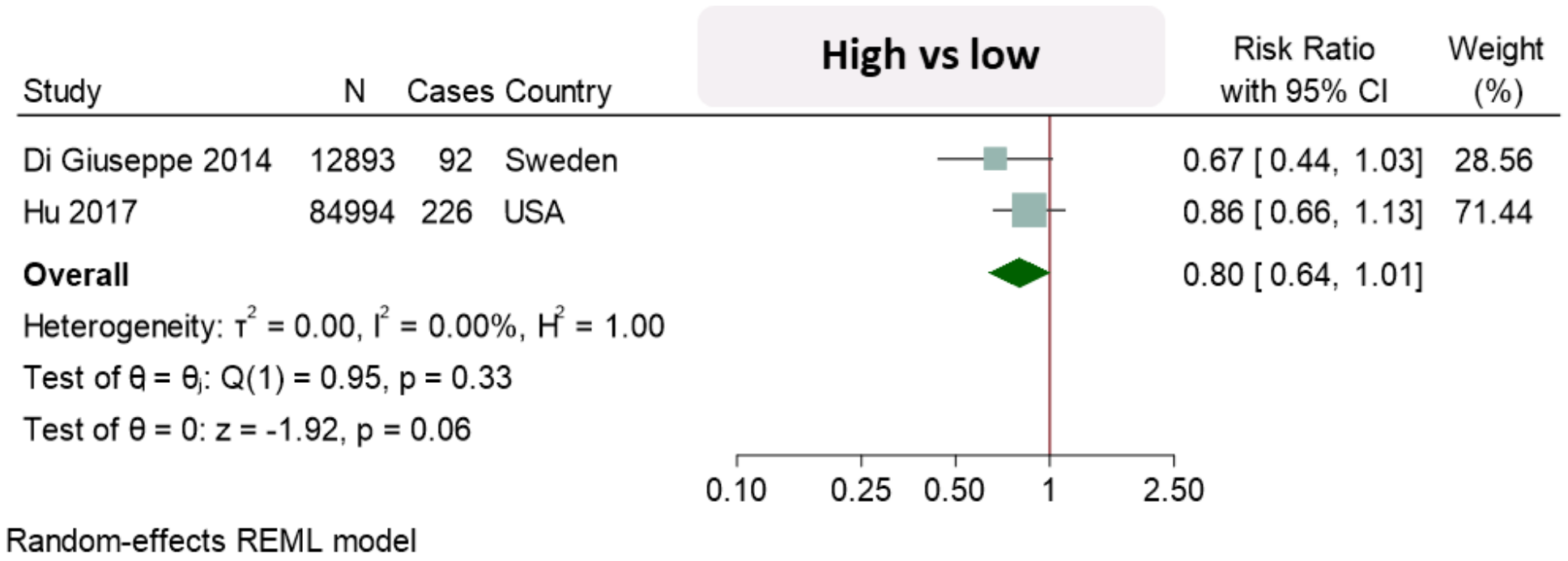

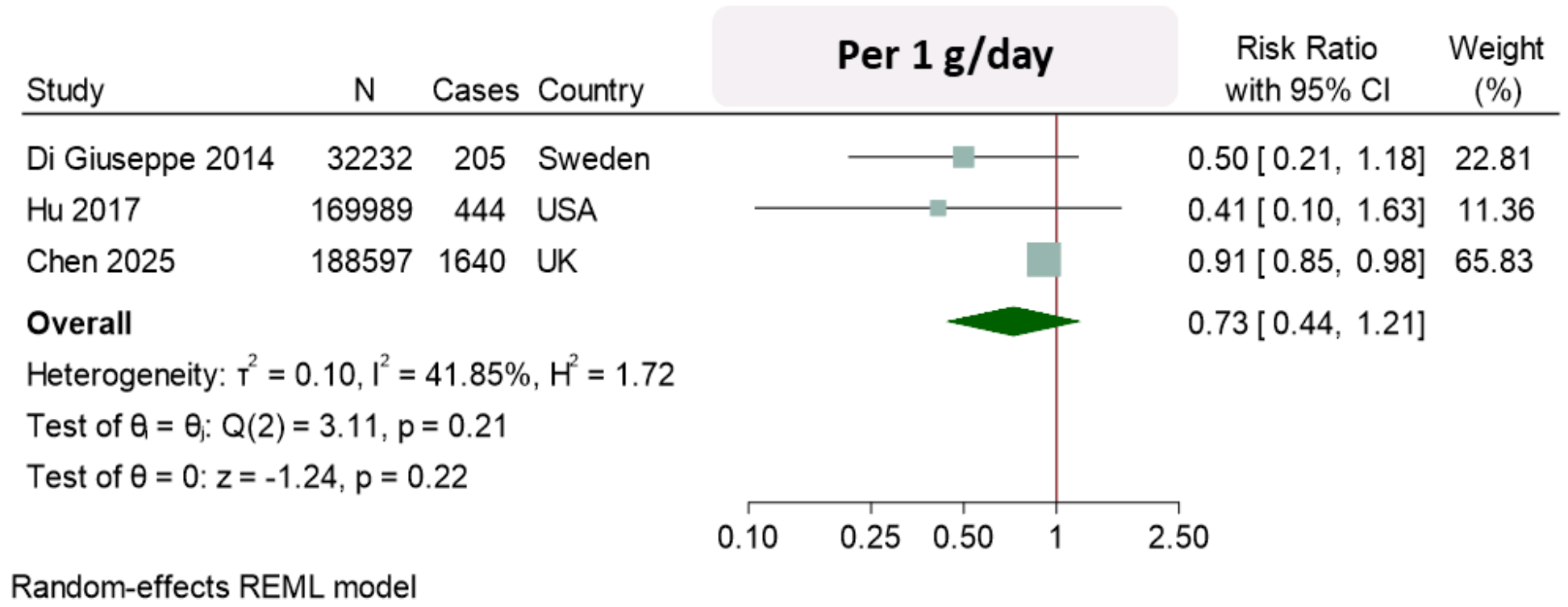

Supplementary Figure 7. Forest plots of risk ratios for rheumatoid arthritis comparing high versus low dietary omega-3 fatty acid intake and per 1 g/day increase, after excluding studies at high risk of bias. CI, confidence interval.

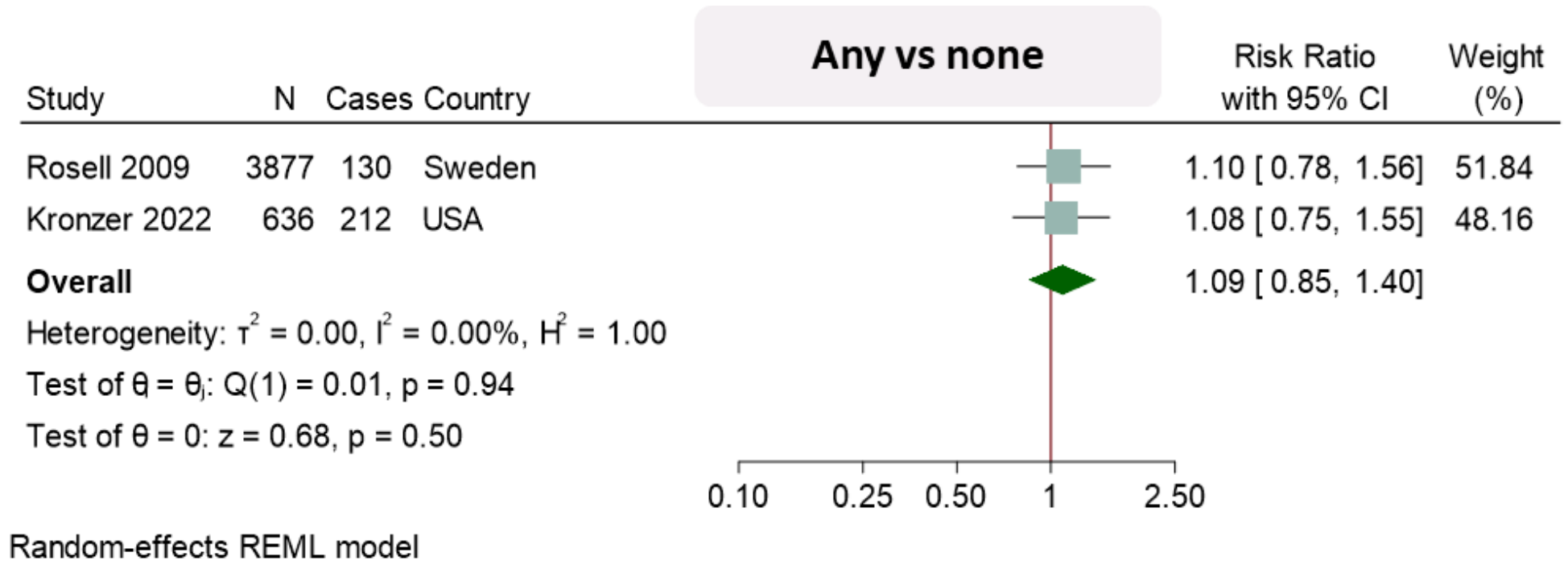

Supplementary Figure 8. Forest plot of risk ratios for rheumatoid arthritis comparing any versus no use of omega-3 fatty supplements. CI, confidence interval.

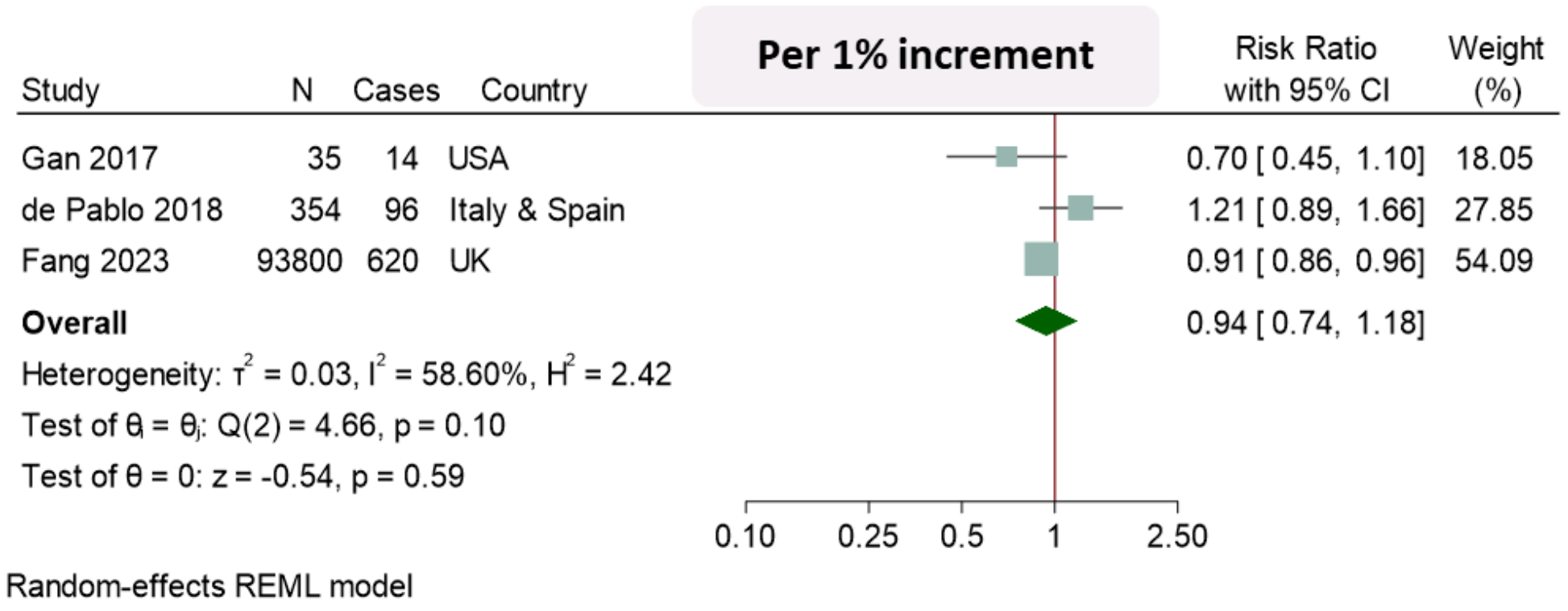

Supplementary Figure 9. Forest plot of risk ratios for rheumatoid arthritis per 1% increment in circulating omega-3 fatty acids. CI, confidence interval.

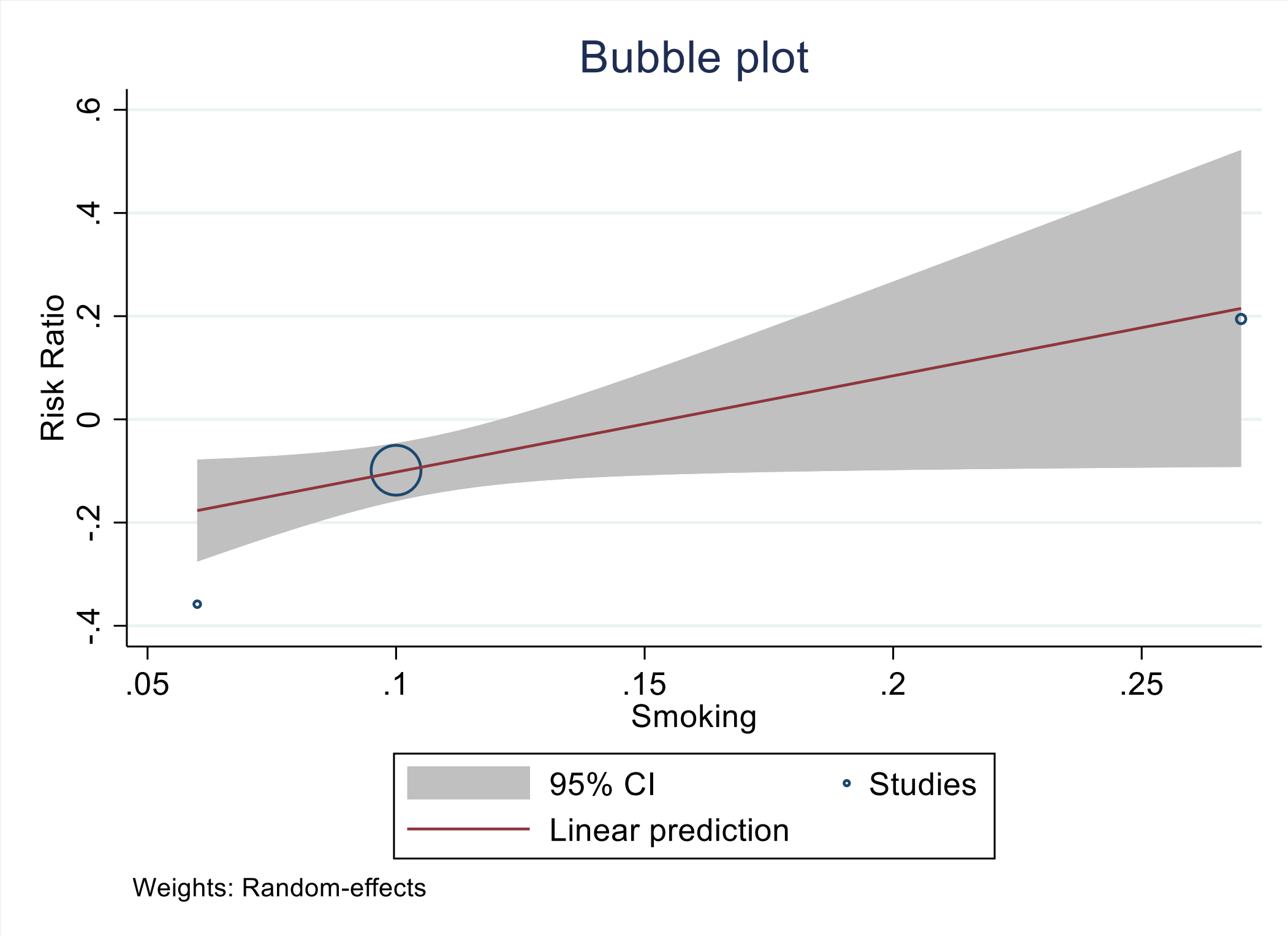

Supplementary Figure 10. Meta-regression of risk ratios for rheumatoid arthritis per 1% increment in circulating omega-3 fatty acids with smoking prevalence within the cohort as effect modifier (*P* = 0.0456). CI, confidence interval.

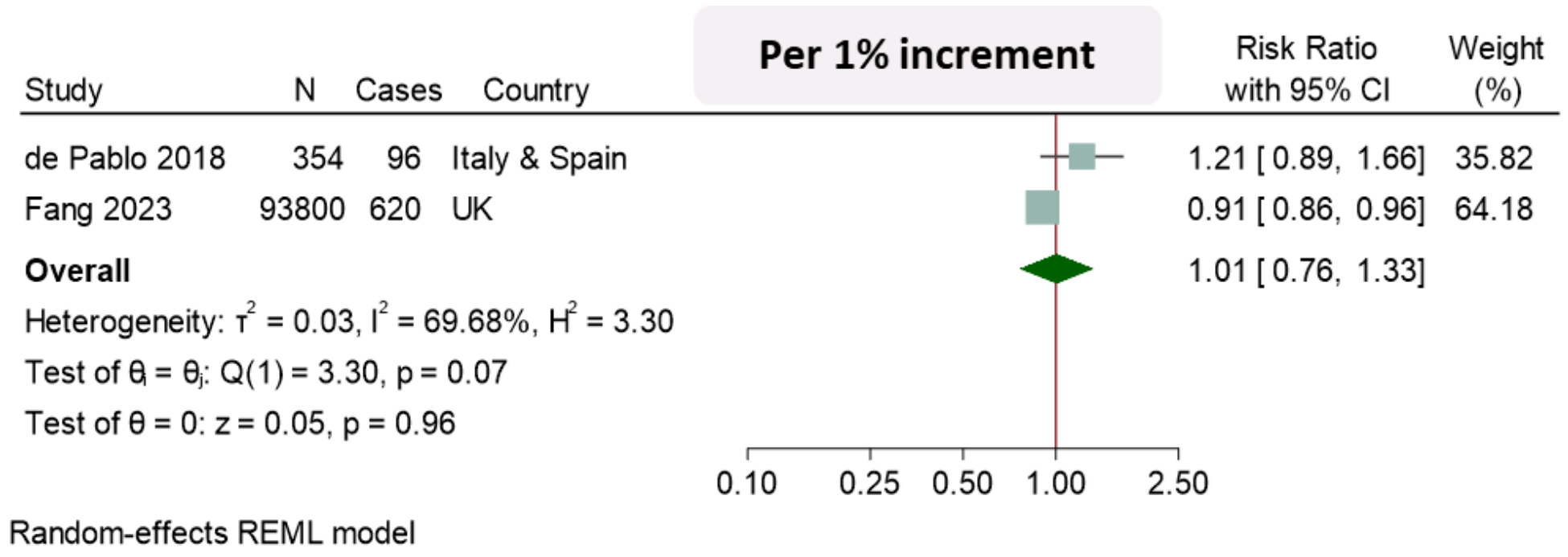

Supplementary Figure 11. Forest plot of risk ratios for rheumatoid arthritis per 1% increment in circulating omega-3 fatty acids, after excluding studies at high risk of bias. CI, confidence interval.

Supplementary references

1. An G, Zhao C, Chen X, Wang W, Bi Y. Casual relationships between circulating metabolites and rheumatoid arthritis: A mendelian randomization study. Heliyon. 2024;10(12):e33085.
2. Hu P, Lee EK-P, Li Q, Tam L-S, Wong SY-S, Poon PK-M, et al. Mediterranean diet and rheumatoid arthritis: A nine-year cohort study and systematic review with meta-analysis. European journal of clinical nutrition. 2025;79(9):888-96.
3. Li J, Xie X, Chen X, Xie L, Luo M, Yin M, et al. Dietary habit and risk of rheumatoid arthritis: a mendelian randomization study identifying protective and risk factors. European journal of nutrition. 2024;64(1):3.
4. Linauskas A, de Thurah A, Andersen GN, Stengaard-Pedersen K. No evidence that diet has any influence on the aetiology of rheumatoid arthritis. Ugeskrift for laeger. 2015;177(16):V08140445.
5. Mizukami Y, Matsui T, Tohma S, Masuko K. Distinct patterns of dietary intake in different functional classes of patients with rheumatoid arthritis. Topics in Clinical Nutrition. 2017;32(2):141-51.
6. Wang Y, Hu S, Zhang W, Zhang B, Yang Z. Dietary factors and rheumatoid arthritis: new perspectives from a Mendelian randomisation analysis. The British journal of nutrition. 2025;133(1):107-17.
7. Zhang J, Fang X-Y, Leng R, Chen H-F, Qian T-T, Cai Y-Y, et al. Metabolic Signature of Healthy Lifestyle and Risk of Rheumatoid Arthritis: Observational and Mendelian Randomization Study. The American journal of clinical nutrition. 2023;118(1):183-93.
8. Ascione S, Barde F, Artaud F, Nguyen Y, Macdonald C, Mariette X, et al. Association between beverage consumption and risk of rheumatoid arthritis: a prospective study from the French E3N Cohort. Rheumatology (Oxford, England). 2023;62(5):1814-23.
9. Duo R, Long X, Bing L, Duan C, Li P, Ma H, et al. Association between dietary inflammatory potential and rheumatoid arthritis: Evidence from the NHANES 2003 to 2018. Medicine. 2025;104(31):e43518.
10. Feng J, Pan C, Gou Y, Yang X, Cheng S, Wei W, et al. EAT-Lancet Diet Modifies the Risk of Rheumatoid Arthritis Through Metabolomic Signature. Arthritis & rheumatology (Hoboken, NJ). 2025(101623795).
11. Gao T, Chen Z-Y, Li T, Tang J-D, Lin X, Hu H-G, et al. The relationship between the composite dietary antioxidant index and rheumatoid arthritis risk in American adults: the mediating role of BMI. Frontiers in immunology. 2025;16(101560960):1560570.
12. Gao Z, Alishahi F, Faridpour A, Nezamoleslami S, Nezamoleslami S, Ghiasvand R, et al. The association between dietary inflammatory index with risk rheumatoid arthritis: A case-control study. Prostaglandins & other lipid mediators. 2024;175(9808648, c3p):106916.
13. Ghaseminasabparizi M, Nazarinia MA, Akhlaghi M. Adherence to the dietary approaches to stop hypertension dietary pattern and rheumatoid arthritis in Iranian adults. Public health nutrition. 2021;24(18):6085-93.
14. Gomez-Vaquero C, Nolla JM, Fiter J, Ramon JM, Concustell R, Valverde J, et al. Nutritional status in patients with rheumatoid arthritis. Joint bone spine. 2001;68(5):403-9.
15. Hahn J, Malspeis S, Choi MY, Stevens E, Karlson EW, Lu B, et al. Association of Healthy Lifestyle Behaviors and the Risk of Developing Rheumatoid Arthritis Among Women. Arthritis Care and Research. 2023;75(2):272-6.
16. Harooni J, Pourjavid A, Amirkhizi F, Hamedi-Shahraki S, Kiani F, Zolghadrpour M-A, et al. Combined healthy lifestyle score and odds of rheumatoid arthritis in Iranian adults: a nested case-control from PERSIAN Dena Cohort Study (PDCS). BMJ open. 2025;15(3):e097715.
17. Jandari S, Mosalmanzadeh N, Shadmand Foumani Moghadam MR, Soleimani D, Shivappa N, Hebert JR, et al. Dietary inflammatory index and healthy eating index-2015 are associated with rheumatoid arthritis. Public health nutrition. 2021;24(18):6007-14.
18. Johansson K, Askling J, Alfredsson L, Di Giuseppe D. Mediterranean diet and risk of rheumatoid arthritis: a population-based case-control study. Arthritis research & therapy. 2018;20(1):175.
19. Junior DST. Environmental and individual factors associated with protection and predisposition to autoimmune diseases. International journal of health sciences. 2020;14(6):13-23.
20. Malani K, Pradhan S, Madani MM, Roberts MB, Shadyab A, Allison M, et al. Association of Diet Quality With Risk of Incident Rheumatoid Arthritis in the Women's Health Initiative. Journal of the Academy of Nutrition and Dietetics. 2024;124(11):1451-73.
21. Meng Q, Dong S, Ge J, Qin C, Zhang G, Fu C, et al. Association between composite dietary antioxidant index and rheumatoid arthritis: results from NHANES 2003-2018. International journal of medical sciences. 2025;22(5):1184-93.
22. Mosalmanzadeh N, Jandari S, Soleimani D, Shadmand Foumani Moghadam MR, Khorramrouz F, Araste A, et al. Major dietary patterns and food groups in relation to rheumatoid arthritis in newly diagnosed patients. Food science & nutrition. 2020;8(12):6477-86.

23. Nezamoleslami S, Ghiasvand R, Feizi A, Salesi M, Pourmasoumi M. The relationship between dietary patterns and rheumatoid arthritis: a case-control study. Nutrition & metabolism. 2020;17(101231644):75.
24. Pang E, Chen X, Zhu Y, Shi L, Wang T, Kou Y, et al. The association between the American heart association's new "Life's Essential 8" and different types of arthritis: insights from a large population study. Scientific reports. 2025;15(1):24319.
25. Rajkumar C, Baghel AS, Shubhangi K, Bhagavathi NNL. Association of Family History, Life-Style Related Factors, Dietary Patterns, and Psychological Status with Amavata (~Rheumatoid Arthritis) among the Population of Jamnagar, India: A Matched Case-Control Study. Journal of Natural Remedies. 2023;23(2):441-54.
26. Rubin KH, Rasmussen NF, Petersen I, Kopp TI, Stenager E, Magyari M, et al. Intake of dietary fibre, red and processed meat and risk of late-onset Chronic Inflammatory Diseases: A prospective Danish study on the "diet, cancer and health" cohort. Int J Med Sci. 2020;17(16):2487-95.
27. Sparks JA, Barbhaiya M, Tedeschi SK, Leatherwood CL, Tabung FK, Speyer CB, et al. Inflammatory dietary pattern and risk of developing rheumatoid arthritis in women. Clinical rheumatology. 2019;38(1):243-50.
28. Surowiec I, Arlestig L, Rantapaa-Dahlqvist S, Trygg J. Metabolite and Lipid Profiling of Biobank Plasma Samples Collected Prior to Onset of Rheumatoid Arthritis. PloS one. 2016;11(10):e0164196.
29. Wu L, Wang J, Wang X, Yue X. Association between life's essential 8 and risk of rheumatoid arthritis: a cross-sectional study. BMC rheumatology. 2025;9(1):72.
30. Xiang S, Wang Y, Qian S, Li J, Jin Y, Ding X, et al. The association between dietary inflammation index and the risk of rheumatoid arthritis in Americans. Clinical rheumatology. 2022;41(9):2647-58.
31. Zhao H, Bai Y, Liu Y, Xing Y, Yan Y, Chen G, et al. Association of ultraprocessed food consumption with risk of rheumatoid arthritis: a retrospective cohort study in the UK Biobank. The American journal of clinical nutrition. 2024;120(4):927-35.
32. Zheng T, Jingchun H, Yimiao L, Zhijun Z, Nan Z, Lan W. Dietary Patterns and Common Diseases in Chinese Elderly: Exploring Associations and Providing Guidance for Personalized Interventions. Current Research in Nutrition and Food Science. 2023;11(3):1263-81.
33. Zhu Y, Garcia-Larsen V, Bromage S, Martinez-Steele E, Curi-Hallal AL, Rebholz CM, et al. Association Between Ultraprocessed Food Intake and Self-Reported Arthritis. American journal of preventive medicine. 2025;68(6):1109-19.
34. Tsoukalas D, Fragoulakis V, Sarandi E, Docea AO, Papakonstaninou E, Tsilimidos G, et al. Targeted Metabolomic Analysis of Serum Fatty Acids for the Prediction of Autoimmune Diseases. Frontiers in molecular biosciences. 2019;6(101653173):120.
35. Dainty J, Sayers E, Yates M, Macgregor A. Pro-inflammatory diets are associated with increased C-reactive protein and rheumatoid arthritis in the UK biobank cohort. 2020;79(SUPPL 1):882-3.
36. Hahn J, Cook N, Alexander E, Friedman S, Bubes V, Walter J, et al. Vitamin D and Marine n-3 Fatty Acid Supplementation and Prevention of Autoimmune Disease in the VITAL Randomized Controlled Trial: Abstract Number: 0957. Arthritis & Rheumatology. 2021;73:1996-8.
37. Costenbader K, Cook N, Lee IM, Hahn J, Walter J, Bubes V, et al. OP0038 VITAMIN D AND MARINE n-3 FATTY ACID SUPPLEMENTATION FOR PREVENTION OF AUTOIMMUNE DISEASE IN THE VITAL RANDOMIZED CONTROLLED TRIAL: OUTCOMES OVER 7 YEARS. Annals of the Rheumatic Diseases. 2022;81(Suppl 1):27-8.
38. Paul B, Pariyapurath R. Risk factor assessment of rheumatoid arthritis in North Kerala. Eur J Rheumatol. 2018;5(3):184-90.
39. Rambod M, Nazarinia M, Raieskarimian F. The impact of dietary habits on the pathogenesis of rheumatoid arthritis: a case-control study. Clin Rheumatol. 2018;37(10):2643-8.
40. Backlund R, Bergstrom U, Compagno M, Arvidsson L, Rydell E, Sonestedt E, et al. Quantified intakes of key diet components and the risk of developing rheumatoid arthritis: Results from a nested case-control study. Annals of the rheumatic diseases. 2025(0372355, 62w).
41. Chen L, Tan T, Chen Y, Cui F, Chen H, Zhao Y, et al. Dietary Polyunsaturated Fatty Acid and the Risk of Rheumatoid Arthritis: Insights From Genetic Predisposition and Proteomics. Arthritis & rheumatology (Hoboken, NJ). 2025(101623795).
42. de Pablo P, Romaguera D, Fisk HL, Calder PC, Quirke A-M, Cartwright AJ, et al. High erythrocyte levels of the n-6 polyunsaturated fatty acid linoleic acid are associated with lower risk of subsequent rheumatoid arthritis in a southern European nested case-control study. Annals of the rheumatic diseases. 2018;77(7):981-7.
43. Di Giuseppe D, Wallin A, Bottai M, Askling J, Wolk A. Long-term intake of dietary long-chain n-3 polyunsaturated fatty acids and risk of rheumatoid arthritis: a prospective cohort study of women. Annals of the rheumatic diseases. 2014;73(11):1949-53.

44. Fang XY, Zhang J, Qian TT, Gao P, Wu Q, Fang Q, et al. Metabolomic profiles, polygenic risk scores and risk of rheumatoid arthritis: a population-based cohort study in the UK Biobank. RMD Open. 2023;9(4).
45. Gan RW, Bemis EA, Demoruelle MK, Striebich CC, Brake S, Feser ML, et al. The association between omega-3 fatty acid biomarkers and inflammatory arthritis in an anti-citrullinated protein antibody positive population. Rheumatology (Oxford, England). 2017;56(12):2229-36.
46. Hatami E, Aghajani M, Pourmasoumi M, Haeri F, Boozari B, Nezamoleslami S, et al. The relationship between animal flesh foods consumption and rheumatoid arthritis: a case-control study. Nutrition journal. 2022;21(1):51.
47. He J, Wang Y, Feng M, Zhang X, Jin Y-B, Li X, et al. Dietary intake and risk of rheumatoid arthritis-a cross section multicenter study. Clinical rheumatology. 2016;35(12):2901-8.
48. Hu Y, Sparks JA, Malspeis S, Costenbader KH, Hu FB, Karlson EW, et al. Long-term dietary quality and risk of developing rheumatoid arthritis in women. Annals of the rheumatic diseases. 2017;76(8):1357-64.
49. Krok-Schoen JL, Brasky TM, Hunt RP, Rohan TE, Baker TA, Li W, et al. Dietary Long-Chain n-3 Fatty Acid Intake and Arthritis Risk in the Women's Health Initiative. Journal of the Academy of Nutrition and Dietetics. 2018;118(11):2057-69.
50. Kronzer VL, Lennon RJ, Sparks JA, Myasoedova E, Davis JM, Crowson CS. Association between work physical activity, dietary factors, and risk of rheumatoid arthritis. Seminars in arthritis and rheumatism. 2022;57(1306053, umv):152100.
51. Linos A, Kaklamanis E, Kontomerkos A, Koumantaki Y, Gazi S, Vaiopoulos G, et al. The effect of olive oil and fish consumption on rheumatoid arthritis--a case control study. Scandinavian journal of rheumatology. 1991;20(6):419-26.
52. Linos A, Kaklamani VG, Kaklamani E, Koumantaki Y, Giziaki E, Papazoglou S, et al. Dietary factors in relation to rheumatoid arthritis: a role for olive oil and cooked vegetables? The American journal of clinical nutrition. 1999;70(6):1077-82.
53. Mazzucca CB, Scotti L, Cappellano G, Barone-Adesi F, Chiocchetti A. Nutrition and Rheumatoid Arthritis Onset: A Prospective Analysis Using the UK Biobank. Nutrients. 2022;14(8).
54. Nguyen Y, Salliot C, Mariette X, Boutron-Ruault M-C, Seror R. Fish Consumption and Risk of Rheumatoid Arthritis: Findings from the E3N Cohort Study. Nutrients. 2022;14(4).
55. Pedersen M, Stripp C, Klarlund M, Olsen SF, Tjonneland AM, Frisch M. Diet and risk of rheumatoid arthritis in a prospective cohort. The Journal of rheumatology. 2005;32(7):1249-52.
56. Rosell M, Wesley A-M, Rydin K, Klareskog L, Alfredsson L. Dietary fish and fish oil and the risk of rheumatoid arthritis. Epidemiology (Cambridge, Mass). 2009;20(6):896-901.
57. Shapiro JA, Koepsell TD, Voigt LF, Dugowson CE, Kestin M, Nelson JL. Diet and rheumatoid arthritis in women: a possible protective effect of fish consumption. Epidemiology (Cambridge, Mass). 1996;7(3):256-63.
58. Sparks JA, O'Reilly EJ, Barbhaiya M, Tedeschi SK, Malspeis S, Lu B, et al. Association of fish intake and smoking with risk of rheumatoid arthritis and age of onset: a prospective cohort study. BMC musculoskeletal disorders. 2019;20(1):2.
59. Sundstrom B, Johansson I, Rantapaa-Dahlqvist S. Diet and alcohol as risk factors for rheumatoid arthritis: a nested case-control study. Rheumatology international. 2015;35(3):533-9.
60. Kang T, Xi Y, Qian T, Lu S, Du M, Shi X, et al. Exploring the link between dietary omega-3 and omega-6 fatty acid intake and rheumatoid arthritis risk: NHANES 1999-2020 study. Clinical and experimental rheumatology. 2024;42(9):1720-8.
61. Lee AL, Park Y. The association between n-3 polyunsaturated fatty acid levels in erythrocytes and the risk of rheumatoid arthritis in Korean women. Annals of nutrition & metabolism. 2013;63(1-2):88-95.
62. Nguyen HD, Oh H, Kim M-S. An increased intake of nutrients, fruits, and green vegetables was negatively related to the risk of arthritis and osteoarthritis development in the aging population. Nutrition research (New York, NY). 2022;99(8303331):51-65.
63. Nguyen HD, Oh H, Kim M-S. Higher intakes of nutrients are linked with a lower risk of cardiovascular diseases, type 2 diabetes mellitus, arthritis, and depression among Korean adults. Nutrition research (New York, NY). 2022;100(8303331):19-32.
64. Tu L, Wei F, Song Y, Huang H, Qing L, Luo X, et al. Development and internal validation of a prediction model for rheumatoid arthritis: a case-control study. Scientific reports. 2025;15(1):16620.
65. Xie R, Zhang Y. Association between 19 dietary fatty acids intake and rheumatoid arthritis: Results of a nationwide survey. Prostaglandins, leukotrienes, and essential fatty acids. 2023;188(p04, 8802730):102530.

66. Backlund RT, Drake I, Bergstrom U, Compagno M, Sonestedt E, Turesson C. Adherence to dietary guidelines, and the risk of developing rheumatoid arthritis: results from a nested case-control study. Rheumatology (Oxford, England). 2024;63(2):407-13.
67. Benito-Garcia E, Feskanich D, Hu FB, Mandl LA, Karlson EW. Protein, iron, and meat consumption and risk for rheumatoid arthritis: a prospective cohort study. Arthritis Res Ther. 2007;9(1):R16.
68. Hu Y, Costenbader KH, Gao X, Hu FB, Karlson EW, Lu B. Mediterranean diet and incidence of rheumatoid arthritis in women. Arthritis care & research. 2015;67(5):597-606.
69. Nguyen Y, Salliot C, Gelot A, Gambaretti J, Mariette X, Boutron-Ruault M-C, et al. Mediterranean Diet and Risk of Rheumatoid Arthritis: Findings From the French E3N-EPIC Cohort Study. Arthritis & rheumatology (Hoboken, NJ). 2021;73(1):69-77.
70. Costenbader KH, Cook NR, Lee IM, Hahn J, Walter J, Bubes V, et al. Vitamin D and Marine n-3 Fatty Acids for Autoimmune Disease Prevention: Outcomes Two Years After Completion of a Double-Blind, Placebo-Controlled Trial. Arthritis & rheumatology (Hoboken, NJ). 2024;76(6):973-83.
71. Hahn J, Cook NR, Alexander EK, Friedman S, Walter J, Bubes V, et al. Vitamin D and marine omega 3 fatty acid supplementation and incident autoimmune disease: VITAL randomized controlled trial. BMJ. 2022;376:e066452.
72. Gan RW, Young KA, Zerbe GO, Demoruelle MK, Weisman MH, Buckner JH, et al. Lower omega-3 fatty acids are associated with the presence of anti-cyclic citrullinated peptide autoantibodies in a population at risk for future rheumatoid arthritis: a nested case-control study. Rheumatology (Oxford, England). 2016;55(2):367-76.
73. Gan RW, Demoruelle MK, Deane KD, Weisman MH, Buckner JH, Gregersen PK, et al. Omega-3 fatty acids are associated with a lower prevalence of autoantibodies in shared epitope-positive subjects at risk for rheumatoid arthritis. Ann Rheum Dis. 2017;76(1):147-52.
74. McGuinness LA, Higgins JPT. Risk-of-bias VISualization (robvis): An R package and Shiny web app for visualizing risk-of-bias assessments. Research Synthesis Methods. 2020;n/a(n/a).
